# Structure, phylogeny, and expression of the frizzled-related gene family in the lophotrochozoan annelid *Platynereis dumerilii*

**DOI:** 10.1186/s13227-015-0032-4

**Published:** 2015-12-04

**Authors:** Benjamin R. Bastin, Hsien-Chao Chou, Margaret M. Pruitt, Stephan Q. Schneider

**Affiliations:** Department of Genetics, Development and Cell Biology, Iowa State University, 503 Science Hall II, Ames, IA 50011 USA; Center for Cancer Research, NIH, Bethesda, MD USA; Department of Pediatrics, University of Chicago, Chicago, IL USA

**Keywords:** Frizzled, sFRP, wnt, Beta-catenin, Spiral cleaving, Signaling center, Cell lineage, Lophotrochozoan, Annelid, Polychaete, Asymmetric cell division, Evolution, Phylogeny

## Abstract

**Background:**

Wnt signaling pathways are highly conserved signal transduction pathways important for axis formation, cell fate specification, and organogenesis throughout metazoan development. Within the various Wnt pathways, the frizzled transmembrane receptors (Fzs) and secreted frizzled-related proteins (sFRPs) play central roles in receiving and antagonizing Wnt signals, respectively. Despite their importance, very little is known about the *frizzled*-*related* gene family (*fzs* & *sfrps*) in lophotrochozoans, especially during early stages of spiralian development. Here we ascertain the *frizzled*-*related* gene complement in six lophotrochozoan species, and determine their spatial and temporal expression pattern during early embryogenesis and larval stages of the marine annelid *Platynereis dumerilii*.

**Results:**

Phylogenetic analyses confirm conserved homologs for four frizzled receptors (Fz1/2/7, Fz4, Fz5/8, Fz9/10) and sFRP1/2/5 in five of six lophotrochozoan species. The *sfrp3/4* gene is conserved in one, divergent in two, and evidently lost in three lophotrochozoan species. Three novel *fz*-*related* genes (*fzCRD1*-*3*) are unique to *Platynereis*. Transcriptional profiling and in situ hybridization identified high maternal expression of *fz1/2/7*, expression of *fz9/10* and *fz1/2/7* within animal and dorsal cell lineages after the 32-cell stage, localization of *fz5/8*, *sfrp1/2/5*, and *fzCRD*-*1* to animal-pole cell lineages after the 80-cell stage, and no expression for *fz4*, *sfrp3/4*, and *fzCRD*-*2*, and -*3* in early *Platynereis* embryos. In later larval stages, all *frizzled*-*related* genes are expressed in distinct patterns preferentially in the anterior hemisphere and less in the developing trunk.

**Conclusions:**

Lophotrochozoans have retained a generally conserved ancestral bilaterian *frizzled*-*related* gene complement (four Fzs and two sFRPs). Maternal expression of *fz1/2/7*, and animal lineage-specific expression of *fz5/8* and *sfrp1/2/5* in early embryos of *Platynereis* suggest evolutionary conserved roles of these genes to perform Wnt pathway functions during early cleavage stages, and the early establishment of a Wnt inhibitory center at the animal pole, respectively. Numerous frizzled receptor-expressing cells and embryonic territories were identified that might indicate competence to receive Wnt signals during annelid development. An anterior bias for *frizzled*-*related* gene expression in embryos and larvae might point to a polarity of Wnt patterning systems along the anterior–posterior axis of this annelid.

**Electronic supplementary material:**

The online version of this article (doi:10.1186/s13227-015-0032-4) contains supplementary material, which is available to authorized users.

## Background

Wnt signaling pathways are highly conserved signal transduction pathways that have widespread functions during development in all metazoans including essential roles in cell fate specification, cell proliferation, and embryonic axis formation [1–3]. Three main Wnt pathways have been identified. The Wnt/Ca^2+^ pathway regulates intracellular Ca^2+^ levels [4, 5], the Planar Cell Polarity (PCP) pathway polarizes cells within an epithelial sheet [6], and the canonical Wnt or Wnt/beta-catenin pathway elicits the transcription of target genes. Canonical Wnt/beta-catenin signaling is the most studied of the three Wnt pathways. Central to this pathway is the regulation of beta-catenin stability. Upon pathway activation, degradation of beta-catenin is inhibited and cytoplasmic and nuclear levels of beta-catenin protein rise. High levels of nuclear beta-catenin promote the formation of transcriptional activators, elicit new gene expression, and lead to subsequent specification of cell fates [1, 7–9].

Central to each of the three Wnt pathways are members of the frizzled family of transmembrane receptors and secreted proteins [10–12]. Frizzled receptors, first identified in *Drosophila melanogaster* as factors involved in planar cell polarity [13], are 7-pass transmembrane receptors with an extracellular cysteine-rich domain (CRD) that binds secreted Wnt ligands. This Wnt ligand-frizzled receptor interaction activates the Wnt pathway by transmitting the signal via structural changes to the receptor’s cytoplasmic domain. In the canonical Wnt pathway, this structural change facilitates the association with and inhibition of a beta-catenin destruction complex, and subsequently leads to nuclear accumulation of beta-catenin [1]. In addition to frizzled receptors, a second class of frizzled family genes, the secreted frizzled-related proteins (sFRP), have been identified as modifiers of Wnt signaling. These sFRPs consist of an N-terminal CRD that is evolutionarily related to the CRD of frizzled receptors, and a C-terminal Netrin domain [14, 15]. sFRPs are thought to inhibit Wnt signaling by competitively binding Wnt ligands [16].

Previous phylogenomic analyses have suggested that the last common ancestor of eumetazoans, a clade that includes cnidarians and bilaterians, had a *frizzled*-*related* gene complement consisting of four frizzled receptors and two sFRPs [2, 17, 18]. This ancestral *frizzled*-*related* gene set of six expanded and retracted during vertebrate evolution due to two rounds of whole genome duplication followed by gene loss early in the vertebrate lineage [10, 19, 20]. Today, most vertebrates outside the teleost fish possess ten frizzled receptors and four sFRPs (five in mammals) [10, 21, 22]. These receptors have been numbered Fz1–Fz10, and the sFRPs have been numbered sFRP1–sFRP5. The origin of each can be traced back to one of the ancestral *frizzled* genes, which have been named *fz1/2/7, fz4, fz5/8, fz9/10, sfrp1/2/5*, and *sfrp3/4*. The two closely related *fz3* and *fz6* genes are restricted to vertebrates and are of uncertain evolutionary origin, although some phylogenetic analyses position them close to or within the *fz1/2/7* gene family [17, 18]. Previous studies have determined that *sfrp1/2/5* and *sfrp3/4* are not closely related, and did not originate from one ancestral *sfrp 1/2/3/4/5* gene. Despite having a similar domain structure, a CRD domain linked to a Netrin (NTR) domain, there is strong evidence that both genes likely originated by two independent but similar gene duplication events that generated a fusion of a frizzled-related CRD domain with a NTR domain [17].

While *frizzled*-*related* genes are well studied in vertebrates including mammals, several investigations over the last decade began to examine *frizzled*-*related* genes in a wider range of invertebrate species during early development [23–28]. These studies have mainly focused on *fz1/2/7,fz5/8*, and *sfrp1/2/5,* and revealed similar embryonic expression domains for orthologous genes suggesting evolutionary conserved roles [29–34]. Although functional evidence in invertebrate embryos is scarce, the observation of anterior expression domains of the Wnt antagonist *sfrp1/2/5* in many invertebrate embryos supports an evolutionary conserved role of *sfrp1/2/5* in the establishment of an anterior Wnt inhibitory center in metazoan embryos [3, 35].

To further investigate the presence and expression of the *frizzled*-*related* gene complement in invertebrate species, we focused on lophotrochozoan species, especially the annelid *Platynereis dumerilii*. Lophotrochozoans constitute one of the three major branches of bilaterians and include invertebrate groups like annelids, mollusks, nemerteans, flatworms, and numerous enigmatic smaller invertebrate phyla like brachiopods and bryozoans [36–40]. Several of these phyla exhibit a common mode of early embryogenesis called spiral cleavage, a series of invariant and stereotypic asymmetric cell divisions that generate a spiral arrangement of embryonic cells of distinct size and position along the animal-vegetal axis of the embryo. These phyla have also been traditionally grouped as ‘Spiralia.’ Intriguingly, some recent metazoan phylogenetic studies imply that ‘spiral cleavage’ might even be an ancestral condition making the clade ‘Spiralia’ synonymous with ‘Lophotrochozoa’ [41], while a more recent analysis by Laumer and colleagues suggests that the lophotrochozoans are a subgroup of the spiralians [38].

**Fig. 1:**
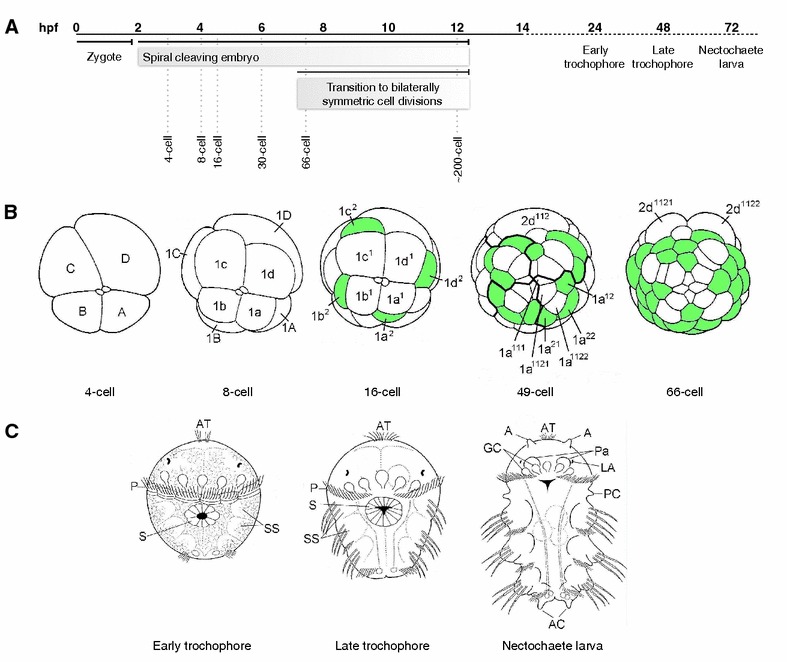
Development stages of *Platynereis dumerilii*. **A** Temporal development of *Platynereis* beginning with fertilization (0 hpf), spiral cleavage stages (2 to 12 hpf), early and late trochophore (24 and 48 hpf), and nectochaete (72 hpf) larval stages. The first cell division begins shortly after 2 hpf, followed by a period of spiral cleavages, and then a transition to a bilaterally symmetrical pattern of cell divisions after 7 hpf. **B** Unequal spiral cleavage pattern of *Platynereis* embryos. Schematics depict animal-pole views of spiral cleavage stages. The *two small circles* in the center of the 4- to 16-cell stages represent the two polar bodies. The 4-cell stage shows the unequal size and nomenclature of the four quadrants/founder cells (*A*–*D*). The 8-cell stage shows the animal-pole 1st micromeres (*1a*, *1b*, *1c*, and *1d*; or *1q*) and their vegetal-pole daughter cells (*1A*, *1B*, *1C*, and *1D*; or *1M*). The 16-cell stage depicts the daughter cell pairs of the first micromeres (*1q* ^*1*^ and *1q* ^*2*^). The 49-cell stage indicates the cell progeny contributed from each quadrant (*dark lines*) and highlights the cleavage pattern of the progeny of the first micromeres indicating the nomenclature for the 1a progeny, the first micromere of the A quadrant. Each of the four quadrants generates one small rosette cell (*1q* ^*111*^) and a larger daughter cell (*1q* ^*112*^) whose progeny will form the anterior head region. These cells form the ‘annelid cross’ (*white*) and are surrounded by cells (*green*) that will form the ciliated ring/prototroch of the larvae. The 66-cell stage highlights the first bilaterally symmetrical cleavage in the 2d cell lineage giving rise to 2d^1121^ and 2d^1122^ cells whose progeny will form the trunk ectoderm. **C** Schematics of three larval stages, the early (24 hpf) and late (48 hpf) trochophore, and the mid nectochaete (72 hpf), ventral views with anterior to the top. The prototroch is a ciliated ring of cells located between the anterior head region/episphere and the posterior trunk region/hyposphere. The episphere harbors the apical organ, eyes, and brain. The trunk region contains the three larval segments including the chaetal sacs. The pygidium includes the posterior growth zone where new segments are added. The stomodeum is located on the ventral side adjacent to the prototroch. Chaetal sacs are three segmental pairs of primordia that give rise to appendages, the parapodia. By the nectochaete stage, parapodia are well established and the head region becomes distinct. Abbreviations: *A* antenna, *AC* anal cirri, *AT* apical tuft, *GC* larval gland cells, *LA* larval eyes, *P* prototroch, *Pa* palps, *PC* peristomial cirrus, *S* stomodeum, *SS* setal (chaetal) sacs. Schematics are modified from Fischer and Dorresteijn 2004 [43] and Pruitt et al. 2014 [54]

Our lophotrochozoan of choice, the annelid *Platynereis dumerilii*, exhibits a typical mode of unequal spiral cleavage during early embryogenesis (Fig. 1) [42–44]. The first two cell divisions are highly unequal giving rise to four large embryonic cells of different sizes, the two smaller A and B cells, one larger C cell, and the largest D cell (Fig. 1B, 4-cell stage). These founder cells or quadrants are ordered alphabetically in a clockwise direction when viewed from the animal pole marked by a pair of polar bodies. The next cell division of each founder cell is oriented along the animal-vegetal axis giving rise to smaller animal-pole daughter cells, the first micromeres 1a, 1b, 1c, and 1d and the larger vegetal-pole daughter cells, the macromeres 1A, 1B, 1C, and 1D forming the 8-cell stage. Each micromere is shifted clockwise with respect to its sister macromere. During the next cell division, the four first micromeres in each quadrant 1a, 1b, 1c, and 1d (1q) divide along the animal-vegetal axis tilted counterclockwise giving rise to a larger animal-pole daughter cell named 1q^1^ and a smaller vegetal-pole daughter cell 1q^2^, with the 1q^1^ cells shifted counterclockwise with respect to the more vegetally localized 1q^2^ sister cells (Fig. 1B, 16-cell stage). The four macromeres 1A, 1B, 1C, and 1D (1M) divide similarly along the animal-vegetal axis forming the animal-pole daughter cells 2a, 2b, 2c, and 2d (2q) shifted counterclockwise in relation to their vegetal-pole daughter cells 2A, 2B, 2C, and 2D (2M). This pattern of cleavage continues with alternating clockwise and counterclockwise shifts leading to a spiral arrangement of cells when viewed from the animal pole.

By the ~49-cell stage (Fig. 1B), the progeny of the four 1q^11^ cells in each of the four quadrants has generated four small characteristic animal-pole daughter cells, the rosette cells (1q^111^), at the animal pole, and four larger vegetal-pole sister cells (1q^112^), the dorsal and ventral cephaloblasts. The cephaloblasts have divided once more to generate 1q^1121^ and 1q^1122^ sister cell pairs (white cells) in each quadrant that form the ‘annelid cross’ surrounded by cells (green) that will form a ciliated ring, the prototroch. The rosette cells will later contribute to the apical organ, and the cephaloblasts will form most of the head region including eyes and brain of the annelid trochophore larvae [42, 44].

Of special significance is the larger D quadrant in *Platynereis* embryos that will generate two extremely large founder cells, the 2d^112^, a progeny of the 2nd ‘micromere’ 2d, and the 4th ‘micromere’ 4d, the mesentoblast, that will give rise to the trunk ectoderm and mesoderm, respectively. These large founders cells are the first to switch from spiral cleavage to a mode of cleavage that generates a bilateral symmetrical arrangement of progeny [42, 44–46].

By 24-h post fertilization (hpf), *Platynereis* has developed into an early trochophore larvae (Fig. 1C), with the prototroch fully formed separating the anterior head or episphere from the posterior trunk region or hyposphere [43, 44, 47]. The prototroch is used for locomotion and comprises a ring of multiciliated cells circumnavigating the embryo. It persists throughout the trochophore stages but begins to disappear by the nectochaete stage (~3-day-old larvae). In the head region of the trochophore larva, the ciliated apical organ, brain, and other anterior structures have formed from the progeny of the rosette cells (1q^111^) and their sister cells, the 1q^112^ cells. The stomodeum anlage, the future mouth, becomes visible on the ventral side of the early trochophore larva adjacent to the prototroch. Posterior to the anus in the hyposphere, the pygidium has formed. By late trochophore stage (~48 hpf), the three trunk segments are visible, each containing a pair of primordia, the chaetal sacs, which will give rise to the bristle (chaetae) bearing parapodia, the appendages of the annelid. On either side of the ventral midline, bilaterally symmetric ciliated structures called paratrochs begin to form posterior to each segment. After 3 days of development, a distinctive head region begins to emerge and becomes increasingly separate from the trunk. At this nectochaete larval stage, the segmental appendages/parapodia including elongated chaetae are fully formed and take over functions in locomotion [43, 47].

Previous work indicated that canonical Wnt/beta-catenin signaling is essential in early *Platynereis* development [48]. During the transition from the 4-cell to the 8-cell stage, strong nuclear localization of beta-catenin protein can be observed in the four vegetal-pole macromeres (1 M), while the four animal-pole micromeres (1q) are lacking any nuclear beta-Catenin. This suggests that the Wnt/beta-catenin pathway is activated in the macromeres. During most subsequent cell divisions through the ~220-cell stage, the asymmetric localization pattern of beta-catenin is repeated, suggesting that every vegetal-pole daughter cell exhibits activated canonical Wnt signaling, whereas the animal-pole daughter cells do not. Indeed, this asymmetric beta-catenin activation acts as a binary cell fate switch. Inhibition of the beta-catenin degradation complex with the drug 1-Azakenpaullone leads to global beta-catenin nuclear localization, and to animal-pole daughter cells adopting the cell fate of their vegetal-pole daughter cells [48]. Similar beta-catenin-mediated binary switches have now been found in all three major branches of bilateral symmetrical animals, although restricted to nematode, ascidian, and annelid embryos with fixed stereotypic, invariant cell lineages [49–52]. However, the molecular mechanism causing this asymmetric pattern in early *Platynereis* embryos remains unknown. While the full complement of Wnt ligands has been surveyed comprehensively in both early and late *Platynereis* development [53, 54], it is not yet known whether and which frizzled receptors might be involved. As expression and function of the larger *frizzled*-*related* gene family are largely unexplored in any lophotrochozoan species, especially during early spiral embryogenesis, we decided to investigate the *frizzled*-*related* gene family in embryos and larvae of *Platynereis*.

Here we present the first comprehensive look at the *frizzled*-*related* gene family in lophotrochozoans, and determine the *frizzled*-*related* gene complement in six lophotrochozoan species. Using an RNA-seq time course spanning the first 14 h of *Platynereis* development, we have identified nine *frizzled*-*related* genes in this annelid species and have quantified their stage-specific expression. Analyses of structural features and phylogeny identified well-conserved orthologous genes for four Frizzled receptors, *fz1/2/7, fz5/8, fz9/10*, and *fz4* and one conserved sFRP, *sfrp1/2/5*, two derived *sfrp3/4*-like genes, and two novel *frizzled*-*related* genes with similarities to sFRPs. Using whole-mount in situ hybridization, we have determined the spatial expression patterns of seven *frizzled*-*related* genes in early embryos and larval stages. This comprehensive study of *frizzled* expression in *Platynereis* embryos and larvae suggests numerous Wnt signaling inputs into annelid development, and indicates evolutionary conserved functions for *fz1/2/7, fz5/8*, and *sfrp1/2/5* in patterning early embryos. Furthermore, the presented work provides the critical information necessary for a functional dissection of Wnt signaling in this species.

## Methods

### *Platynereis dumerilii* culture

*Platynereis* embryos and larvae were obtained from a breeding culture at Iowa State University maintained according to protocols available at http://www.platynereis.de [43, 54]. Newly fertilized eggs were placed in an 18 °C incubator to ensure constant temperature throughout early development.

### Transcriptome assembly

After incubation at 18 °C, embryos were collected at 2, 4, 6, 8, 10, 12, and 14 hpf with biological replicates, homogenized in Trizol (Ambion), and stored at −80 °C before RNA was extracted according to manufacturer’s protocol. RNA was treated with RNase-free DNase Set (QIAGEN) prior to purification with RNeasy Mini Kit (QIAGEN). Deep sequencing with 75 bp-100 bp paired-end reads was performed at Duke Institute for Genome Sciences and Policy using an Illumina HiSeq sequencing system. Reads were filtered with Trimmomatic [55] and assembled de novo using the Trinity method [56]. Expression levels were calculated in FPKM (fragments per kilobase per million mapped reads) using the RSEM software package [57]. To compare the expression level across samples, we used a scaling normalization method called TMM (trimmed mean of *M* values) [58] to get the TMM-normalized FPKM.

### Alignment and phylogenetic analysis

*P. dumerilii* sequences were derived from RNA-seq data and verified by cloning and Sanger sequencing. Sequences from other species were obtained from NCBI and JGI databases (see Additional file 1: Table S1; Additional file 2: Table S2). Representative species were chosen from each of the major phylogenetic groups including chordates (*H. sapiens, X. laevis, D. rerio, B. floridae*), echinoderms (*S. purpuratus*), hemichordates (*S. kowalevskii*), ecdysozoans (*D. melanogaster, C. elegans, T. castaneum, D. pulex*), lophotrochozoans (*P. dumerilii, C. gigas, C. teleta, A. californica, H. robusta, L. gigantea*), and a nonbilaterian (*N. vectensis*). Frizzled family genes were identified by reciprocal BLAST using well-annotated queries from *H. sapiens*. Conserved domains were identified using NCBI Batch Web-CD Search Tool. Sequences of conserved domains were aligned with Mafft [59] using the Mafft iterative approach (L-INS-i) for maximum speed and accuracy [60]. Multiple alignments were visualized and manually edited in Aliview [61]. Positions that consisted of 70 % or more gaps were removed. Phylogenetic analysis was performed using Mr. Bayes [62] with the InvGamma model of substitution rates. Analysis ran for 2,000,000 generations with a 500,000 generation burn in. Smoothened sequences were used as an outgroup for CRD tree, and TIMP sequences were used as an outgroup for the NTR tree. Trees were visualized in FigTree (http://tree.bio.ed.ac.uk/software/figtree/) and modified for publication in Adobe Illustrator. Highly divergent species and sequences were removed before final CRD analysis (see Additional file 1: Table S1).

### Cloning of Frizzled family genes

Sequences for Frizzled receptors and sFRPs were obtained from the assembled transcriptome, and primers were designed using Primer3 [63]. Primers used were as follows: *fz1/2/7* full ORF clone, forward: GCATGTCTTGATTGGAGTCG, reverse: TTGATGAGTGATGATTTGTCAAC; *fz4* full ORF clone, forward: CTTTGCACCTCAGTGACACA, reverse: AACGAGGGCCATAAATCTTG: *fz5/8* full ORF clone, forward: CTCCAGCCCCTATTTCAACA, reverse: GTCTTCCCTGACCAGATCCA; *fz5/8* partial clone, forward: CTCCAGCCCCTATTTCAACA, reverse: GTCTTCCCTGACCAGATCCA; *fz9/10* partial clone, forward: TGTCCTCAGCTGTGACAACC, reverse: GTTTCTCGAACTTGCGAAGG: *sfrp1/2/5* full ORF clone, forward: TTGTGAAAGGTGACTGTTAAACG, reverse: CATTAGTCCATTGAGATTACTTTTCG; *sfrp1/2/5* partial clone, forward: TACCAACCGAAGTGTGTGGA, reverse: TTGTCTCCCTTCCTGTTTCG; *sfrp3/4* full ORF clone, forward: TTGCTGCTGCTATGTGAAGG, reverse: GCTGATGGAGCTTCTTTCCA; and *fzCRD*-*1* full ORF clone, forward: TCCAAAATGAAGAGCCTTGTG, reverse: GCAGCCTCCAAAGGTAAGG. Target sequences were PCR amplified using Standard Taq Polymerase (New England Biolabs) and ligated into pGEM-T Easy vector (Promega), except for *fz4* and *sfrp3/4* which were amplified using OneTaq (New England Biolabs) and ligated into PCR II Dual Promoter vector (Invitrogen). Plasmid DNA was isolated using Plasmid Mini Kit (Qiagen), and sequences were verified by Sanger sequencing with T7 and Sp6 primers. Sequences for *P. dumerilii frizzled1/2/7, frizzled4, frizzled5/8, frizzled9/10, sfrp1/2/5, sfrp3/4*, and *fzCRD*-*1* were deposited in GenBank with accession numbers KT989648-KT989654.

### Whole-mount in situ hybridization

Templates for probe synthesis were generated from plasmid DNA linearized with an appropriate restriction enzyme to result in a probe of ~1000 nucleotides. Antisense RNA probes were synthesized using Sp6 (Roche) or T7 (New England Biolabs) RNA polymerase kits and DIG RNA labeling mix (Roche). Embryos >18 hpf were fixed in a solution of 4 % paraformaldehyde 0.1 M MOPS free acid, 2 mM EGTA, 1 mM MgSO_4_, and 0.1 %Tween-20 for at least 4 h on a nutator at 4 °C. Embryos <18 hpf were treated in a solution of 50 mM Tris, 495 mM NaCl, 9.6 mM KCl, 27.6 mM Na_2_SO_4_, 2.3 mM NaHCO_3_, and 6.4 mM EDTA at pH 8.0 two times for 3 min prior to fixation to remove the vitelline membrane [48]. Embryos were then fixed overnight on a nutator at 4 °C. Whole-mount in situ hybridization was performed according to previously published protocols [64] with previously described modifications [54]. Embryos were stored at 4 °C in PBT for up to 2 weeks to reduce background before staining with DAPI (4′,6-diamidino-2-phenylindole; Sigma). Embryos were mounted in 87 % glycerol and stored at 4 °C. Embryos <16 hpf were imaged on a LSM700 Microscope with an AxioCam MRc5. Older embryos and larvae were imaged with a Zeiss Axioskop 2 microscope with a Canon EOS Rebel T3 camera. Images were adjusted in Adobe Photoshop for brightness and contrast. False color images were generated by first modifying DIC images in Adobe Photoshop and then merging with DAPI images.

## Results

### Identification and phylogenetic analysis of Frizzled family genes in *Platynereis* and other lophotrochozoans

In order to identify and elucidate the *frizzled*-*related* gene family during *Platynereis* development, RNA-seq was performed from RNA collected at early embryonic stages. De novo transcriptome assembly using Trinity software [56] and subsequent annotation by various BLAST-based bioinformatics pipelines identified nine gene models encoding Fz-related cysteine-rich domains (CRDs). These gene models corresponded to four *frizzled* transmembrane receptors, two *sfrps*, and three novel *frizzled*-*related* genes coding for proteins consisting of a frizzled-like CRD domain only, named Frizzled-related CRD 1, 2, and 3 (FzCRD-1, -2, -3). Sequences for all frizzled family gene models were further confirmed using preliminary genomic sequencing data for *Platynereis* (*Platynereis* sequencing consortium and the Arendt laboratory at EMBL, data not shown). Additionally, full-length cDNA clones were established by gene-specific PCR from stage-specific cDNA for six of the seven gene models, while for the seventh, *fz9/10*, a partial fragment (~1000 bp) of the open reading frame was cloned.

### The bilaterian *frizzled*-*related* gene complement

Previous phylogenetic analyses have suggested that the pre-bilaterian ancestor likely had four Frizzled receptors (Fz1/2/7, Fz4, Fz5/8, and Fz9/10) and two sFRPs (sFRP1/2/5 and sFRP3/4) [17, 18]. This conclusion was reached with limited searches within lophotrochozoan/spiralian taxa [17] and excluded sequences for sFRPs [18]. To refine the analysis, frizzled-related sequences from *Platynereis*, other lophotrochozoan/spiralian species with sequenced genomes (the annelids *Capitella telata* and *Helobdella robusta*; the mollusks *Lottia gigantea,Crassostrea gigas*, and *Aplysia californica*) [65–68], and from other phylogenetically informative metazoan taxa were collected and subjected to various phylogenetic analyses (see Additional file 1: Table S1 for complete list; see “Methods” for details).

**Fig. 2:**
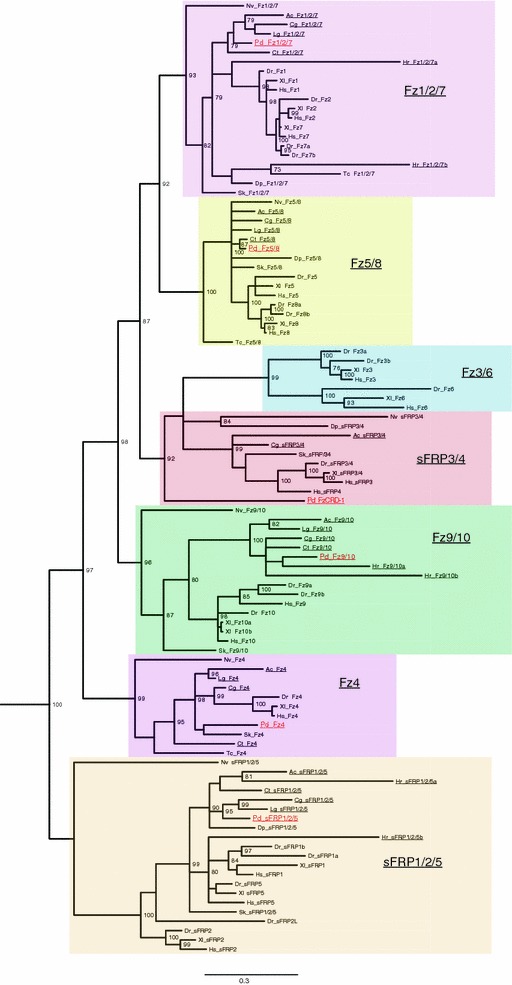
Phylogenetic analysis of Frizzled-related Cysteine-rich domains identifies a conserved lophotrochozoan gene complement. Cysteine-rich domains (CRDs) of frizzled transmembrane receptors and secreted frizzled-related proteins were aligned with MAFFT and analyzed with Mr Bayes. Posterior probabilities greater than 70 % are shown. The CRD of smoothened was used as an outgroup (not shown). Groupings of frizzled and sFRP subfamilies are highlighted with *colored boxes*. *Platynereis dumerilii* proteins are highlighted in *red font*, and cluster within frizzled subgroups with high posterior probability. Lophotrochozoan/Spiralian frizzled-related proteins are *underlined*. The novel *Platynereis dumerilii* protein FzCRD-1 clusters with the sFRP3/4 and vertebrate specific Fz3/6 subgroups with high posterior probability. The *scale bar* indicates the estimated number of substitution per site. The highly derived CRDs of *Platynereis dumerilii* sFRP3/4, FzCRD-2, and -3 were removed from this analysis. Species abbreviations: Ac, *Aplysia californica;* Cg, *Crassostrea gigas;* Ct, *Capitella teleta;* Dp, *Daphnia pulex;* Dr, *Danio rerio;* Hr, *Helobdella robusta;* Hs, *Homo sapiens;* Lg, *Lottia gigantea;* Nv, *Nematostella vectensis;* Pd, *Platynereis dumerilii;* Sk, *Saccoglossus kowalevskii;* Tc, *Tribolium castaneum;* Xl, *Xenopus laevis*

In agreement with previous studies [17, 18], our phylogenetic analysis based on alignments of the CRD domains strongly supports an ancestral *frizzled*-*related* gene complement consisting of four frizzled receptors, *fz1/2/7, fz4, Fz5/8, and fz9/10*, and two sFRPs, *sfrp1/2/5* and *sfrp3/4* (Fig. 2). Consistent with previous evidence [17], our analysis suggests independent evolutionary origins of the two ancestral *sfrp* genes. Under this scenario, the *sfrp1/2/5* gene originated from a domain fusion after duplication of a Frizzled CRD domain and a NTR domain prior to the diversification of the Frizzled receptors. sFRP3/4, which clusters strongly within the frizzled receptors, was proposed to arise from a similar but separate and later domain fusion event. Phylogenetic analysis of NTR domain-containing proteins including the sFRP NTR domains supports this scenario of independent origins (Additional file 1: Table S1; Additional file 3: Figure S1). The sFRP3/4 cluster is far apart from the sFRP1/2/5 cluster within the NTR tree. Contrary to previous studies [17, 18], our analysis did not find support for a close relationship between the chordate specific *fz3/6* and *fz1/2/7* genes. Instead we found a cluster consisting of Fz3/6 and sFRP3/4 that forms a sister group to Fz1/2/7 and Fz5/8. It should be noted that the two previous studies that found a close relationship between Fz1/2/7 and Fz3/6 either had low support for this particular node [17], or did not include sFRP sequences in their analysis [18]. One cannot say with any confidence whether the differing relationships supported in our and previous studies are an artifact of the limited phylogenetic signal within the CRD domain or if our analysis indeed indicates a more complicated evolutionary relationship between Fz3/6 and the other frizzled receptors.

### The lophotrochozoan *frizzled*-*related* gene complement

Of the six lophotrochozoan species included in our study (Fig. 2; Table 1; Additional file 1: Table S1), five (*Aplysia californica, Capitella teleta, Crassostrea gigas, Lottia gigantea*, and *Platynereis dumerilii*) possess well-conserved orthologous genes for five of the six ancestral *frizzled*-*related* genes (*fz1/2/7, fz4, fz5/8, fz9/10*, and *sfrp1/2/5*). The notable exception is *Helobdella robusta*, whose modified gene set suggests the loss of *fz4* and *fz5/8* and duplication of *fz1/2/7, fz9/10*, and *sfrp1/2/5.* Orthologs for the remaining *frizzled*-*related* gene, *sfrp3/4*, are either absent or strongly divergent in 5 of the 6 lophotrochozoans (Fig. 2; Table 1; Additional file 1: Table S1; Additional file 2: Table S2; Additional file 3: Figure S1). Indeed, a previous study indicated that annelids and mollusks might have lost an orthologous *sfrp3/4* gene based on its absence in the *Capitellateleta* and *Lottia gigantea* genomes [17]. However, in our analysis, we were able to confirm that the mollusk *Crassostrea gigas* possesses an *sfrp3/4* gene with well-conserved CRD and NTR domains (Fig. 2; Additional file 3: Figure S1). *Platynereis dumerilii* also has a bona fide *sfrp3/4* that can be identified by its well-conserved NTR domain despite its highly divergent CRD domain. In addition, we have identified genes consisting of only a CRD domain that cluster strongly with other *sfrp3/4* genes in both *Platynereis dumerilii* (*fzCRD*-*1*) and *Aplysia californica*, although the lack of a NTR domain in the latter may be due to an incomplete gene model.

**Table 1:**
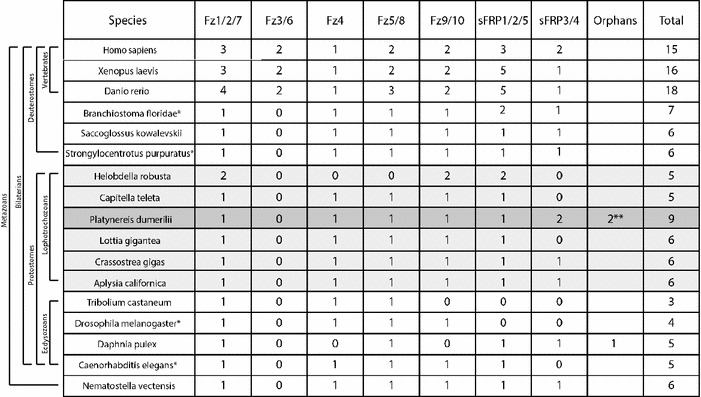
The *frizzled*-*related* gene complement in metazoans

Frizzled-related subfamilies are named on the top. Animal clades/subgroups are indicated with brackets to the left of the ‘species’ column. Each column lists the number of identified *frizzled*-*related* genes in each subgroup within each species

Orphans are additional highly divergent *frizzled*-*related* genes that cannot be placed within one of the six subfamilies

Number of all *frizzled*-*related* genes for each species is listed in the column on the left. Lophotrochozoans are highlighted in light gray. *Platynereis* is highlighted in dark gray

Annotations for *fz9/10* in *D. melanogaster* and *C. elegans* are from Schenkelaars et al. [18]

* See explanation in Additional File 1

** refers to FzCRD-2 and FzCRD-3

### The *Platynereis frizzled*-*related* gene complement

Our phylogenetic analysis confirms that *Platynereis* retained single, well-conserved orthologs of the four ancestral Frizzled receptors and *sfrp1/2/5,* with each one clustering strongly with their respective class of *frizzled*-*related* genes. The exception to this is the divergent *sfrp3/4* gene, which contains a highly derived CRD domain linked to a conserved NTR domain (Additional file 3: Figure S1), and was therefore removed from our CRD analysis. Of the three *fzCRD*-*1,* -*2*, and -*3* genes unique to *Platynereis*, one, *fzCRD*-*1,* clusters with the *sfrp3/4* gene family (Fig. 2) suggesting a more recent evolutionary origin by a duplication of a CRD domain of a *sfrp3/4* gene. This hypothetical event may have also contributed to the divergence of the CRD domain of *Platynereissfrp3/4*. The CRD domains of *fzCRD*-*2* and -*3* are highly derived, and while they do cluster with *frizzled*-*related* genes, they do not cluster reliably within any of the six ancestral *frizzled*-*related* gene families (data not shown). Thus, we speculate that these genes arose from one of the six ancestral *frizzled*-*related* genes by duplication of the CRD only. Confirmed by transcriptome and genomic data, *fzCRD*-*2* and -*3* are expressed at later larval stages, and only at very low levels in early stages (Additional file 4: Table S3; data not shown). Thus, *fzCRD*-*2* and -*3* were not further included in our study.

### Structure of the frizzled-related proteins in *Platynereis*

For six of the seven *frizzled*-*related* genes in *Platynereis,* cDNA clones covering the full coding region were generated. The exception is *fz9/10*, of which a 944 bp fragment coding for the C-terminal end of the CRD and most of the transmembrane domain was cloned. However, the full protein sequence model is confirmed by preliminary genomic data, obtained from the *Platynereis* sequencing consortium and the Arendt laboratory at EMBL (data not shown) and a partial *Platynereis* Fz9/10 protein sequence in GenBank covering the CRD and the N-terminal end of the transmembrane domain [GenBank:AHI16256]. Thus, we have confidence in each of our frizzled family gene models, enabling a structural analysis of the encoded predicted frizzled-related proteins.

**Fig. 3:**
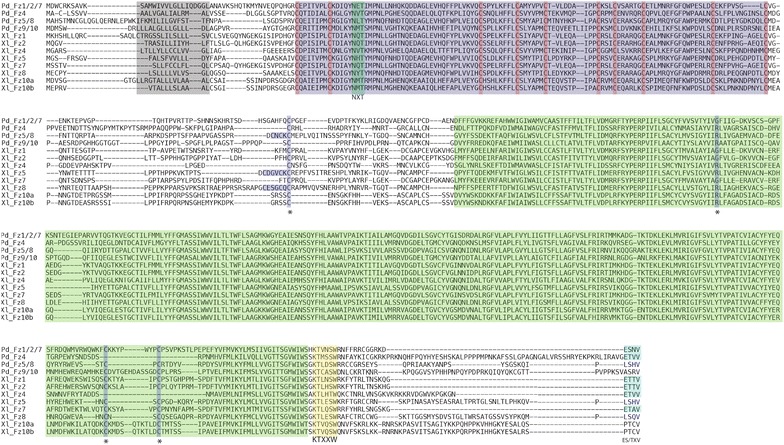
Conserved structural features of *Platynereis* Frizzled transmembrane receptors. Multiple alignments of full-length protein sequences for frizzled transmembrane receptors from *Platynereis dumerilii* (Pd) and *Xenopus laevis* (Xl) using MAFFT are shown. *Gray box* N-terminal hydrophobic localization signal. *Purple box* CRD domain showing conserved Cysteine residues in *orange*, and NXS/T motif in *green*. *Green box* Frizzled transmembrane domain. *Purple boxes* with* asterisks*: conserved ‘signature’ residues unique to each frizzled receptor class identified by Schenkelaars et al. (2012) [18], (Fz1/2/7: 1C–G–2C, Fz4: 1C–R–0C, Fz5/8: 3C–R–2C, Fz9/10: 1C–R–2C). *Yellow box* KTXXXW, PDZ binding motif. *Blue box* C-terminal ES/TXV motif of Fz1/2/7 and Fz4 homologs

**Fig. 4:**
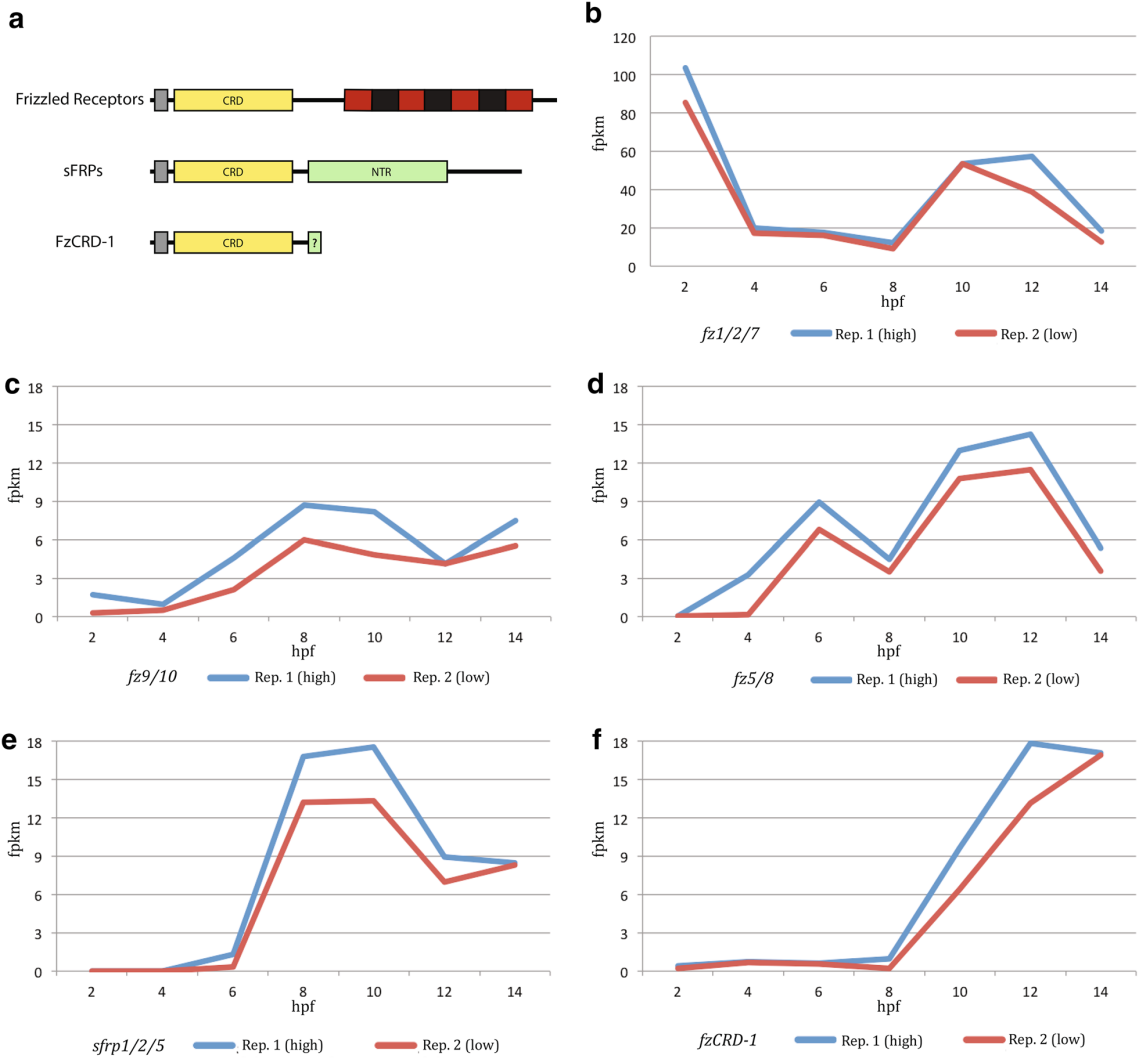
Structure and early temporal expression of *frizzled*-*related* genes in *Platynereis.* **a** Structural domains of the *Platynereis* frizzled receptors, sFRPs, and FzCRD-1. N-terminal *gray boxes* denote membrane localizing signal peptides. *Yellow boxes* are CRDs and *green boxes* are NTR domains. *Black* and *red box* is the seven-pass frizzled transmembrane domain. *Small green box* on FzCRD-1 indicates possible N-terminal remnant of a NTR domain. *Black lines* indicate poorly conserved regions. **b**–**f** Temporal expression of *frizzled*-*related* genes during early *Platynereis* development. **b** *fz1/2/7*, (C) *fz9/10*, **d** *fz5/8*, **e** *sfrp1/2/5*, and **f** *fzCRD*-*1*. The plots illustrate the relative expression levels in FPKM (fragment per kilobase per million mapped reads) based on RNA-seq (Additional file 4: Methods and Results). *X* axis: developmental time in hours post fertilization (hpf). *Y* axis: FPKM. Two biological replicates are shown for each graph with the replicate with the higher FPKM value at each time point (Rep. 1) denoted by *blue lines* and the replicate with lower values at each time point (Rep. 2) denoted by *red lines*

The four conserved frizzled receptor genes *fz1/2/7*, *fz4*, *fz5/8*, and *fz9/10* encode proteins of 568aa, 603aa, 571aa, and 594aa length, respectively (Figs. 3, 4a). Each frizzled receptor protein possesses a N-terminal membrane localizing signal peptide rich in hydrophobic residues followed by an extracellular CRD that contains 10 highly conserved signature cysteine residues [69]. In addition, each Frizzled receptor contains a conserved NXT/S potential glycosylation site exactly six residues after the second cysteine residue. This motif is common to all Frizzled transmembrane receptors and may play a role in Wnt ligand binding [21]. The conserved CRD domains are connected via poorly conserved linker regions to moderately conserved seven-pass transmembrane domains. Each *Platynereis* frizzled receptor retains signature amino acid residues in the linker region and transmembrane domains that are unique to each of the four Frizzled receptor classes that were identified in a recent study [18]. Each Frizzled receptor protein also contains an intracellular conserved KTXXXW motif two residues after the seventh transmembrane domain. This motif has been shown to facilitate Wnt signaling by binding to the PDZ domain of Dishevelled [70]. PdFz1/2/7 and PdFz4 have a conserved ES/TXV motif at the C-terminal end. This motif is found only in Fz1/2/7 and Fz4 orthologs in other species, and has been shown in vertebrates to interact with APC and Discs Large [71].

**Fig. 5:**
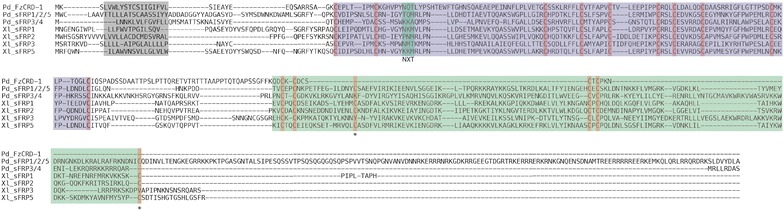
Conserved structural features of *Platynereis* sFRPs. Multiple alignments of full-length protein sequences for sFRPs from *Platynereis dumerilii* (Pd) and *Xenopus laevis* (Xl) using MAFFT are shown. *Gray box* N-terminal hydrophobic localization signal. *Purple box* CRD domain showing conserved Cysteine residues in *orange*, and NXS/T motif in *green*. *Green box* NTR domain with conserved Cysteine residues in *orange*. *Asterisks* indicate sFRP1/2/5 specific Cysteine residues

The two *sfrp* genes in *Platynereis,sfrp1/2/5* and *sfrp3/4*, encode proteins of 458aa and 313aa length, respectively (Figs. 4a, 5). Both proteins have an N-terminal hydrophobic membrane localizing signal peptide followed by a CRD domain. While the sFRP1/2/5 CRD is conserved, the sFRP3/4 CRD is highly divergent and is not recognized as sFRP3/4, although it contains all of the 10 ‘signature’ cysteine residues [69]. As is the case with other sFRP1/2/5 orthologs [21], *Platynereis* sFRP1/2/5 does not have an NXT/S glycosylation site after the second cysteine residue. Unlike other sFRP3/4 orthologs, the highly derived CRD domain of *Platynereis* sFRP3/4 also lacks this motif. Both sFRP1/2/5 and sFRP3/4 contain conserved C-terminal NTR domains.

The novel *fzCRD*-*1* gene in *Platynereis* encodes for a protein of 208aa in length, and consists of a CRD domain only (Figs. 4a, 5). It retains an N-terminal signal peptide rich in hydrophobic residues, indicating it is likely secreted like the sFRPs. The CRD domain in FzCRD-1 is highly conserved and all 10 ‘signature’ cysteine residues are present. Unlike *Platynereis* sFRP3/4, FzCRD-1 possesses an NXT/S glycosylation site after the second cysteine residue that is also found in other species’ sFRP3/4 and all Frizzled receptors [21]. C-terminal to the CRD domain is a variable region linked to a motif that might be a N-terminal fragment of a NTR domain. This short sequence retains a CXC motif that is conserved at the N-terminal end of NTR domains of sFRP3/4 proteins, in contrast to the CXXC motif found in the NTR of sFRP1/2/5 proteins. Thus, both the structural features of FzCRD-1 and the phylogenetic analysis of its CRD domain support the scenario that this gene originated by duplication of the N-terminal domain of an *sfrp3/4* gene. Due to the presence of a membrane localization signal peptide and a highly conserved CRD domain, it is tempting to speculate that FzCRD-1 may also be involved in Wnt ligand binding either to sequester and antagonize Wnt signals extracellularly or perhaps modify the signal in other ways. In fact, a protein of similar structure is produced as a splice variant of *fz4* in vertebrates. This splice variant introduces a stop codon immediately after the region coding for the CRD domain, producing a variant protein that has been shown to both positively and negatively regulate Wnt signaling depending on the cellular context [72].

### *Frizzled*-*related* genes during early *Platynereis* embryogenesis

As Frizzleds play a central role in receiving and modulating Wnt signaling, and Wnt/beta-catenin signaling has been shown to be essential for a global and reiterative binary specification module acting throughout early *Platynereis* development [48], we wanted to know which *frizzled*-*related* transcripts are present in early stages. To do so we determined the temporal expression of *frizzled*-*related* genes by stage-specific transcriptional profiling (RNA-seq). RNA-seq was performed from whole RNA collected at 2-h intervals from the one-cell zygote to a stereogastrula stage: 2 hpf (one-cell zygote), 4 hpf (~8 cells), 6 hpf (~30 cells), 8 hpf (~80 cells), 10 hpf (~140 cells), 12 hpf (~220 cells), and 14 hpf (~330 cells). Subsequent stage-specific quantification of expression levels resulted in transcriptional profiles for each transcript throughout early development (see “Methods”).

Our transcriptional profiling found five of the nine *frizzled*-*related* genes, *fz1/2/7*, *fz5/8*, *fz9/10*, *sfrp1/2/5*, and *fzCRD*-*1* are expressed at significant levels (Fig. 4b–f), and *fz4*, *sfrp3/4*, and *fzCRD*-*2* and-*3* not present at detectable levels within the first 14 h of development (Additional file 4: Table S3). The highest expression levels, measured in fragments per kilobase per million mapped reads (FPKM), were observed for *fz1/2/7* (maternal: ~90; zygotic: ~60), followed by *sfrp1/2/5* and *fzCRD*-*1* (both with zygotic: ~20), *fz5/8* (zygotic: ~15), and *fz9/10* (zygotic: ~10). It should be noted that ‘maternal’ refers to expression levels in the one-cell zygote at 2 hpf. Measurements of the two biological replicates (blue, replicate 1: higher measured level; red, replicate 2: lower measured level) were in good agreement (Fig. 4b–f; Additional file 4: Table S3). Significant maternal expression was only observed for *fz1/2/7* (~ 90) and *fz9/10* (< 2), followed by a dramatic drop in transcript levels for *fz1/2/7* from zygote to 8-cell stage (from 90 to 20), indicating a rapid degradation of this mRNA. The earliest zygotic onset of transcription was observed for *fz5/8* and *fz9/10* between the 8-cell and 30-cell stage (4 to 6 hpf), followed by *sfrp1/2/5* between the 30-cell and 80-cell stage (6 to 8 hpf), and *fzCRD*-*1* after the 80-cell stage (8 to 10 hpf). A strong increase in zygotic expression of *fz1/2/7* and *fz5/8* was also observed after the 80-cell stage (8 to 10 hpf). To confirm the results from transcriptional profiling and to determine the spatial localization of *fz*-*related* transcripts, we determined expression domains by whole-mount in situ hybridization for *fz1/2/7*, *fz5/8*, *fz9/10*, *sfrp1/2/5*, and *fzCRD*-*1* throughout early development.

### Early expression of *Platynereis fz1/2/7*

**Fig. 6:**
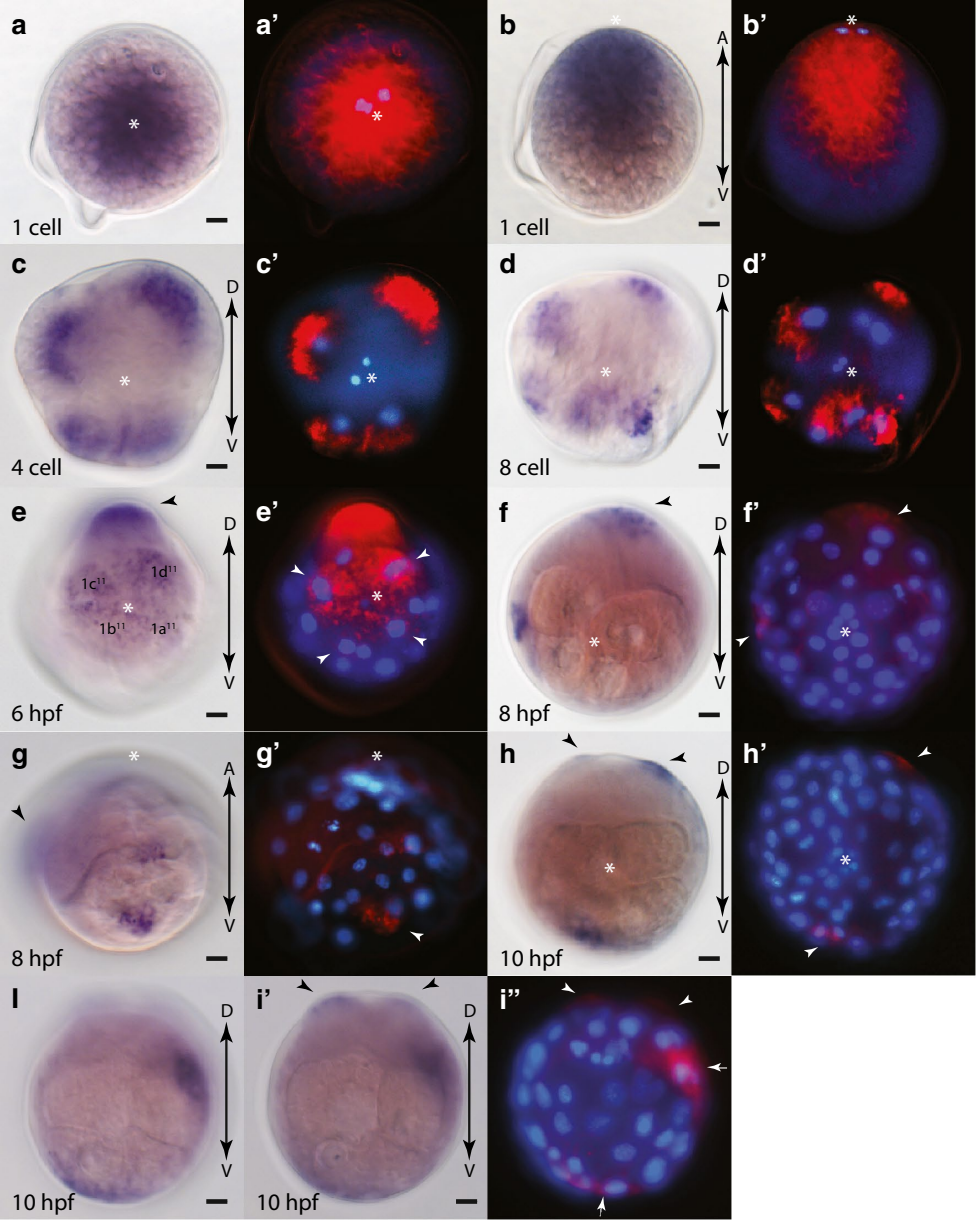
Expression of *fz1/2/7* during early development in *Platynereis.* **a**–**i**′ WMISH of *fz1/2/7,* and **a**′-**i**″ false color images of the WMISH (*red*) overlaid with DAPI-stained nuclear images (*blue*). Animal-pole view (**a**, **a**′) and side view with animal pole up (**b**, **b**′) of 1 cell embryos. 4-cell (**c**, **c**′), 8-cell (**d**, **d**′) embryos, animal-pole view. **e**, **e**′ 6 hpf embryo, animal-pole view. *White arrowheads* point to 1q^11^ cells. 8 hpf embryo animal pole (**f, f**′) and side (**g**, **g**′) views. *White arrowheads* in **f**′ point to expression domains in **c** and **d** quadrants. *White arrowheads* in **g**′ point to expression in **c** quadrant. 10 hpf embryo animal pole (**h**, **h**′) and vegetal pole (**i**, **i**′, **i**″) views. *White arrowheads* in** h**′ point to expression in **d** and **a**/**b** quadrants. *White arrowheads* in **i**″ point to expression in 2d^1121^ and 2d^1122^ progeny. *White arrows* in **i**″ point to expression in **c**, **a** and **b** quadrants. *Asterisks* mark the animal pole. *Black arrows* indicate the orientation of the dorsal–ventral (*D*-*V*) and animal-vegetal (*A*-*V*) axis. *Black arrowheads* in **e**, **f**, **g**, **h**, and **i**′ indicate 2d cell lineage. *White asterisk* in** a**–**h**′ indicates location of animal pole

*In situ* hybridization of one-cell stages confirms a high maternal contribution of *fz1/2/7* revealing that transcripts are concentrated within the clear, yolk-free cytoplasm segregated towards the animal pole of the zygote (Figs. 4b, 6a, a′, b, b′). Transcripts are inherited by each daughter cell after early cleavage divisions forming 4- and 8-cell stage embryos (Fig. 6c, c′, d, d′). At the 30-cell stage (6 hpf), transcripts are enriched in the 2d cell lineage and in the four 1q^11^ cells at the animal pole (Fig. 6e, e’). As the clear cytoplasm of the zygote is preferentially segregated towards these cells [42], this expression may represent the remaining maternal transcripts. By 8 hpf, *fz1/2/7* expression is no longer detectable in the animal-pole cell lineages (1q^11^); however, remaining maternal transcripts or new zygotic expression can be observed within the 2d cell progeny (Fig. 6f, f′). In addition, zygotic expression can be observed in the C quadrant (Fig. 6g, g′) most likely within the 2c lineage. By 10 hpf, areas of expression can be seen within all four quadrants (Fig. 6h–i″). Within the D quadrant (Fig. 6g, g′, h, h′), expression is strongest in the 2d^1121^ and 2d^1122^ cell lineages, in the C quadrant (Fig. 6i, i′, i″) in the 2c cell lineage, and in the A and B quadrant most likely in the 2a and 2b cell lineages, respectively. It should be noted that these are the domains of strongest expression with some weaker ubiquitous expression throughout the whole embryo.

### Early expression of *Platynereis fz9/10*

**Fig. 7:**
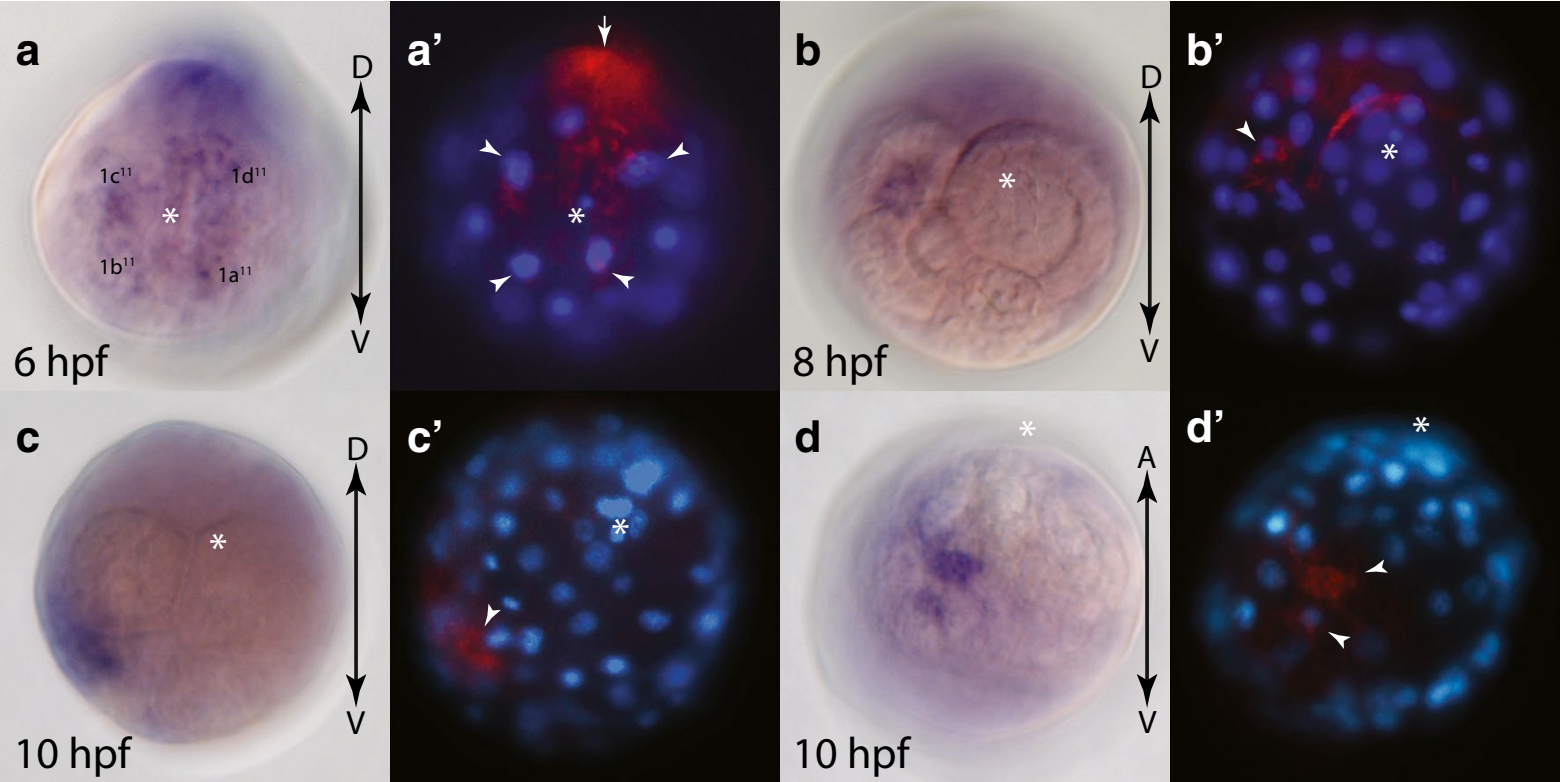
Expression of *fz9/10* during early development in *Platynereis.* **a**–**d** WMISH of *fz9/10,* and **a**′–**d**′ false color images of WMISH (*red*) overlaid with DAPI-stained nuclear images (*blue*). **a**, **a**′ 6 hpf embryo, animal-pole view. *White arrowheads* point to 1q^11^ cells. *Arrow* points to 2d expression. **b**, **b**′ 8 hpf embryo, animal-pole view. *White arrowhead* points to expression in **c** quadrant. 10 hpf embryo, animal pole (**c**, **c**′) and side (**d**, **d**′) views. *White arrowheads* point to **c** quadrant expression. *White asterisks* mark animal pole. *Black arrows* indicate the orientation of the dorsal–ventral (*D*-*V*) and the animal-vegetal (*A*-*V*) axis

While having a minimal maternal contribution (Fig. 4c), the first detectable zygotic expression of *fz9/10* is observed at 6 hpf with enrichments in the 2d cell lineage, and the four animal-pole micromeres 1q^11^ (Fig. 7a, a′) similar to the expression pattern observed for *fz1/2/7* at 6 hpf. At 8 hpf (Fig. 7b, b′) and 10 hpf (Fig. 7c, c′, d, d′), expression is likely confined to the C quadrant, specifically to 2c and its progeny. No stronger expression was observed in the A and B quadrants at these stages.

### Early expression of *Platynereis fz5/8*

**Fig. 8:**
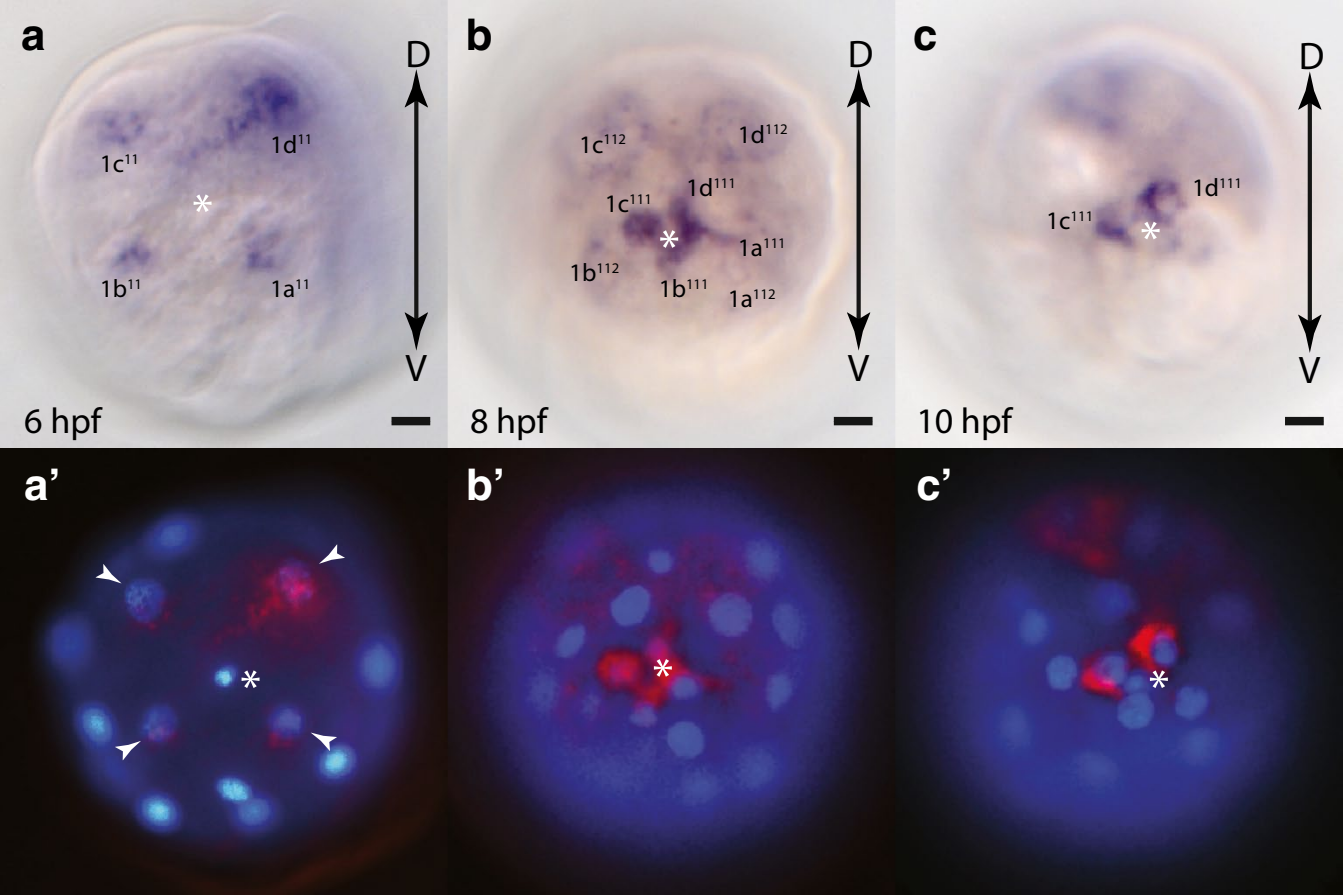
Expression of *fz5/8* during early development in *Platynereis.* **a**–**c** WMISH of *fz5/8,* and **a**′, **c**′ false color images of WMISH (*red*) overlaid with DAPI-stained nuclear images (*blue*). **a**, **a**′ 6 hpf embryo, animal-pole view. *White arrowheads* indicate 1q^11^ cells. 8hpf (**b**, **b**′) and 10 hpf (**c**, **c**′) embryos, animal-pole view. *White asterisks* indicate animal pole. *Black arrows* indicate the orientation of the dorsal–ventral axis (*D*-*V*)

*fz5/8* is first expressed at 6 hpf (Fig. 4d) and is confined to the four animal-pole micromeres, 1q^11^ (Fig. 8a, a′). Between 6 and 8 hpf, the 1q^11^ micromeres divide, each forming one smaller animal-pole daughter cell (1q^111^, the rosette cells) and a larger vegetal-pole daughter cell (1q^112^). At 8 hpf, *fz5/8* expression is observed in all of the progeny of 1q^11^, with the strongest expression in the four rosette cells, and weaker expression in the progeny of 1q^112^ cells, the dorsal and ventral cephaloblasts (Fig. 8b, b′). By 10 hpf, expression is strongest in the two rosette cells of the C and D quadrant, 1c^111^ and 1d^111^, while weaker expression remains in 1a^111^ and 1b^111^, and in progeny of the dorsal cephaloblasts, 1c^112^ and 1d^112^ (Fig. 8c, c′).

### Early expression of *Platynereis sfrp1/2/5*

**Fig. 9:**
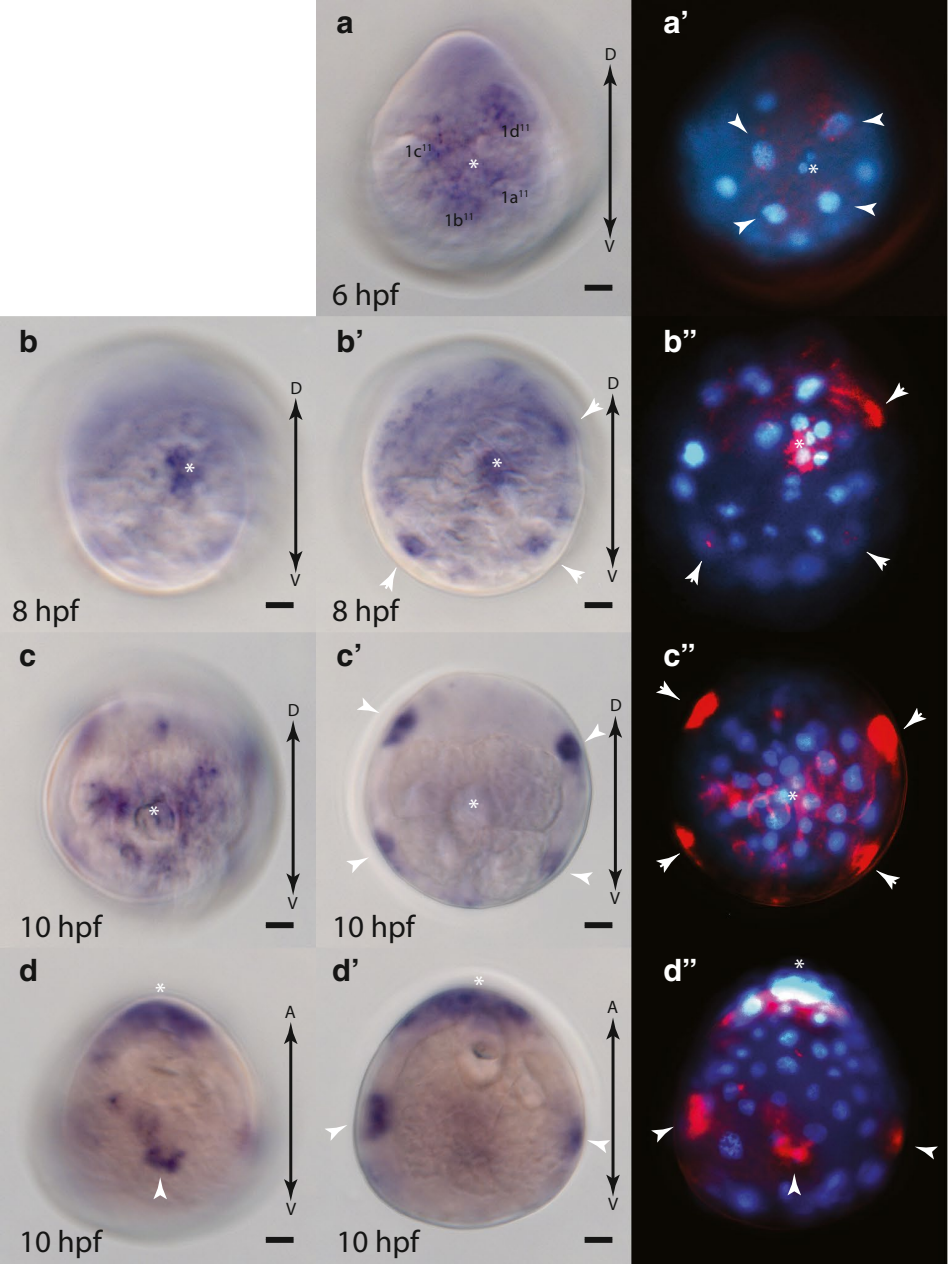
Expression of *sfrp1/2/5* during early development in *Platynereis.* **a**–**d**, **b**′–**d**′ WMISH of *sfrp1/2/5,* and **a**′, **b**″–**d**″ false color images of WMISH (*red*) overlaid with DAPI-stained nuclear images (*blue*). **a**, **a**′ 6 hpf embryo, animal-pole view. White arrowheads indicate 1q^11^ cells. **b**, **b**′, **b**″ 8 hpf embryo focusing on animal pole (**b**) and mid-section (**b**′) of embryo. Expression in rosette cells can be seen at the animal pole in **b** and **b**″. *White arrowheads* indicate more vegetal expression domains in **a**, **b** and **d** quadrants, likely in the 3q lineage. **c** Quadrant expression is not yet distinct. **c**, **c**′, **c**″ 10 hpf embryo with an animal-pole view focusing on animal pole in **c**, and mid-section view in **c**′. Expression throughout 1q^11^ progeny can be seen at animal pole in **c** and **c**″. *White arrowheads* indicate expression in 3q lineage in all four quadrants. **d**, **d**′, **d**″ 10 hpf embryo side view from **c** quadrant showing shallow focus (**d**) and deeper focus (**d**′). **d** Quadrant is to the *left*, and **b** quadrant is to the *right*. *White asterisks* indicate animal pole. *Black arrows indicate* direction of dorsal–ventral (*D*-*V*) and animal-vegetal (*A*-*V*) axis

The Wnt antagonist *sfrp1/2/5* is first expressed around 6 hpf (Fig. 4e) in the four animal-pole micromeres 1q^11^ cells (Fig. 9a, a′). Similar to *fz5/8* expression, *sfrp1/2/5* is expressed strongly in the rosette cells 1q^111^ and less in their sister cells 1q^112^ at 8 hpf (Fig. 9b, b″). In addition to this animal-pole domain, *sfrp1/2/5* is also weakly expressed in one single cell in each quadrant closer to the vegetal pole, the third micromeres 3a, 3b, 3c, and 3d (Fig. 9b′, b″). At 10 hpf, expression remains strong within the animal-pole cell lineages, but unlike *fz5/8*, *sfrp1/2/5* expression is not primarily confined to the rosette cells. Instead similar strong expression is seen throughout the progeny of the dorsal and ventral cephaloblasts (Fig. 9c, c″). At this time, the four expression domains located more vegetally in each quadrant are stronger and more distinct (Fig. 9c′, c″). Lateral views show that each domain entails 2 to 3 individual cells likely the progeny of the 3q lineage (Fig. 9d–d″; Additional file 5: Figure S2). Although not well studied, the 3q lineages are thought to primarily contribute to the formation of ectomesodermal muscles and the stomodeum envelope [45].

### Early expression of *Platynereis fzCRD*-*1*

**Fig. 10:**
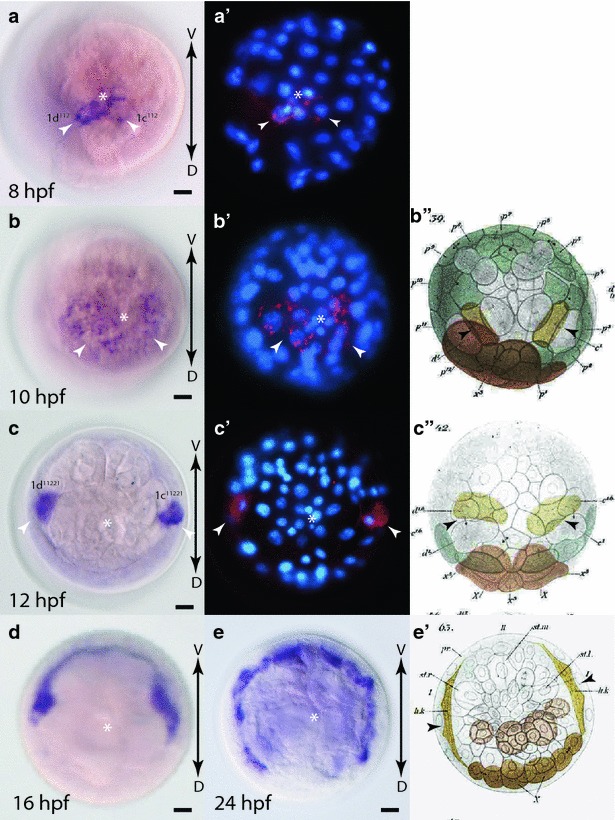
Expression of *fzCRD*-*1* during early development in *Platynereis.* **a**–**e** WMISH of *fzCRD*-*1,* and **a**′–**c**′ false color images of WMISH (*red*) overlaid with DAPI-stained nuclear images (*blue*). **a**, **a**′ 8 hpf embryo, animal-pole view. White arrowheads indicate 1c^112^ and 1d^112^ cells. **b**, **b**′ 10 hpf embryo animal-pole view. *White arrowheads* indicate expression in 1c^112^ and 1d^112^ progeny. **c**, **c**′ 12 hpf embryo animal-pole view. *White arrowheads* indicate expression in 1c^11221^ and 1d^11221^ which have migrated laterally by this point in development. **d** 16 hpf and **e** 24 hpf embryos, animal-pole views showing continued expression in elongating ring canal. **b**″, **c**″, **e**′ Modified images from Wilson (1892) [44] showing migration and elongation of ring canal cells (*yellow cells* indicated by *black arrowheads*). WMISH images are shown with ventral at the *top* to align with Wilson’s original sketches. *White asterisks* indicate animal pole. *Black arrows* show the orientation of the dorsal–ventral axis (*D*-*V*)

The unique *fzCRD*-*1* gene, encoding a CRD domain related to sFRP3/4, has expression beginning at 8 hpf (Fig. 4f). At this stage, *fzCRD*-1 expression is confined to two cells near the animal pole, likely the dorsal cephaloblasts 1c^112^ and 1d^112^ (Fig. 10a, a′). Between 8 and 10 hpf, the dorsal cephaloblasts give rise to three progeny each, and *fzCRD*-*1* is expressed in each of them (Fig. 10b, b′). Two of these cells, 1c^11221^ and 1d^11221^, cease dividing, migrate to the interior, assume a bilaterally symmetric lateral position, and give rise to a circular structure adjacent to the ciliated cells of the prototroch called the ring canal (or ‘head kidney’) first described by Wilson in 1892 [44] (Fig. 10b″, c″, e′). By 12 hpf, *fzCRD*-*1* expression is restricted to these two cells and the expression increases as they ingress and migrate laterally (Fig. 10c, c′). By 16 hpf, these two cells have elongated and are beginning to encircle the inside of the embryo continuing to express *fzCRD*-*1* (Fig. 10d). By 24 hpf, they have almost completely encircled the embryo to form the ring canal (Fig. 10e) [46]. Expression of *fzCRD*-*1* is still visible in the ring canal, although beginning to wane, at 48h-old larval stages (Additional file 6: Figure S3).

### *Frizzled* expression in later *Platynereis* development

**Fig. 11:**
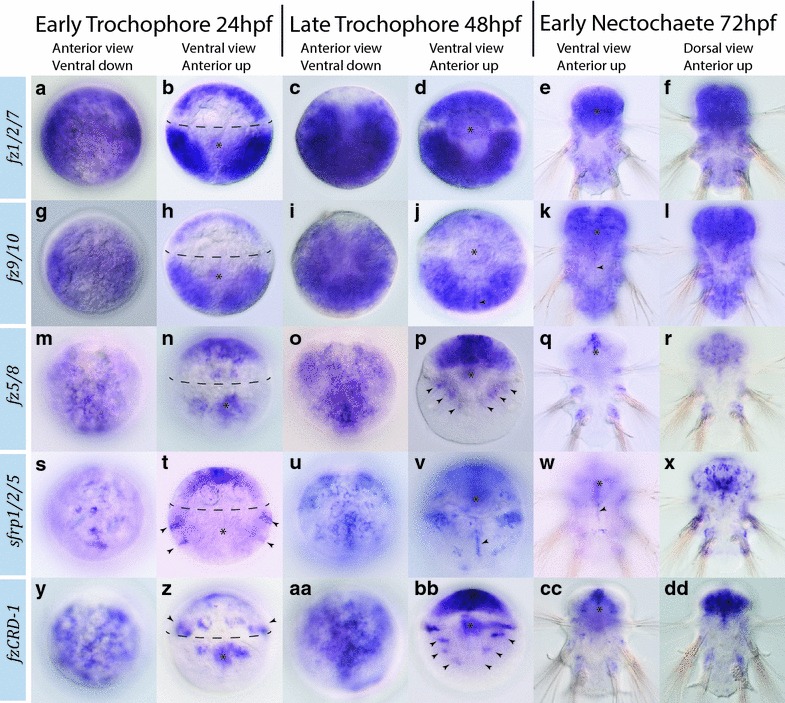
Expression of *fz1/2/7, fz9/10, fz5/8, sfrp1/2/5,* and *fzCRD*-*1* in early (24 hpf) and late (48 hpf) trochopore and nectochaete (3-day old) *Platynereis* larvae. Gene expression analysis was performed with WMISH. Probes are listed on the *left* of each *row*. Stages and orientations are listed at the *top* of each *column*. Refer to the “Results” for details on the expression patterns. *Dotted line* in *B*, *H*, *N*, *T*, and *Z* indicate the location of the ciliated prototroch. *Asterisk* in ventral views indicates the stomodeum. *Black arrowheads* indicate the following: ventral midline expression (*J*, *K*, *V* and *W*), *fz5/8* expression in chaetal sacs (*P*), *sfrp1/2/5* expression in early forming segments (*T*), *fzCRD*-*1* expression in the ring canal (*Z*), and bilaterally symmetric domains in the trunk (*BB*)

Each of the early expressed frizzled family genes, *fz1/2/7*, *fz9/10*, *fz5/8*, *sfrp1/2/5*, and *fzCRD*-*1,* continues to be expressed throughout trochophore and nectochaete larval stages (Fig. 11). *fz1/2/7* and *fz9/10* show similar expression patterns during this period of development. Both are highly expressed throughout ectodermal and mesodermal domains in the epi- and hypospheres at 24 hpf (Fig. 11a, b, g, h), and absent from the ciliated prototroch, presumptive stomodeum and ventral midline. By 48 hpf, expression of both *fz1/2/7* and *fz9/10* can be seen in the stomodeal rosette (Fig. 11d, j), and *fz9/10* also begins to be more prominently expressed at the ventral midline (Fig. 11j). In 3-day-old larvae, both genes remain highly expressed throughout head and trunk ectoderm and *fz9/10* additionally shows expression in ventral and dorsal midline cells (Fig. 11e, f, k, l).

*fz5/8* and *sfrp1/2/5*, both expressed earlier in the 1q^11^ cells and/or their progeny that form the episphere, continue to be expressed in the developing head and brain at 24, 48, and 72 hpf (Fig. 11m–dd). Compared to the even expression of *fz1/2/7* and *fz9/10* throughout the head region, both *fz5/8* and *sfrp1/2/5* transcripts are elevated in distinct subdomains, especially within the most anterior territories that harbor the apical organ. Our results agree with a previous study that reported anterior expression of *fz5/8* and *sfrp1/2/5* in the developing brain and apical organ in early trochophore larvae [35]. In addition, *fzCRD*-*1*, which is confined to the cells of the ring canal in early stages of development, shows anterior expression resembling the expression of *fz5/8* within the episphere at 24 hpf (Fig. 11m, y), and throughout the developing hind- and forebrains at 48 and 72 hpf (Fig. 11o, q, r, aa, cc, dd). There is coexpression of *fzCRD*-*1* and *fz5/8* in the stomodeum at 24 and 48 hpf (Fig. 11n, p, z, bb). *sfrp1/2/5* transcripts are also expressed in the stomodeum at 48 hpf, but not at the earlier larval stage. Unlike *fz5/8* and *fzCRD*-*1*, *sfrp1/2/5* shows a segmental expression pattern in the trunk ectoderm at 24 hpf (Fig. 11t), resembling the expression of *wnt5* at this stage [54]. At 48 hpf, all three genes, *fz5/8*, *fzCRD*-*1*, and *sfrp1/2/5,* are expressed in distinct, non-overlapping domains in the trunk; *fz5/8* is confined to the base of chaetal sacs (Fig. 11p), *sfrp1/2/5* maintains segmental expression in the ectoderm and exhibits additional expression along the ventral midline which is also observed at 72 hpf (Fig. 11v, w), and *fzCRD*-*1* is expressed in three pairs of bilaterally symmetrical domains in the trunk ectoderm at 48 hpf (Fig. 11bb). The bilaterally symmetric expression domains of *fzCRD*-*1* may be the locations of the developing segmental ciliary structures, the paratrochs. *fzCRD*-*1* is also expressed in three bilaterally symmetrical lateral domains on the dorsal side that are likely the site where growing chaetae penetrate the surface ectoderm (Additional file 6: Figure S3).

**Fig. 12:**
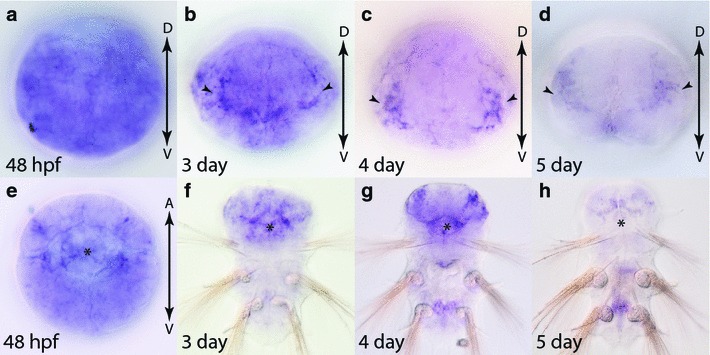
Expression of *fz4* in larval stages (48 hpf to 5-day old) in *Platynereis.* **a**–**d** Anterior views of the head region with ventral side down. **e**–**h** Ventral views with anterior side up. *Asterisks* indicate the stomodeum. *Black arrowheads* indicate specific staining in brain. Gene expression analysis was performed with WMISH. Refer to the “Results” for details on the expression patterns

**Fig. 13:**
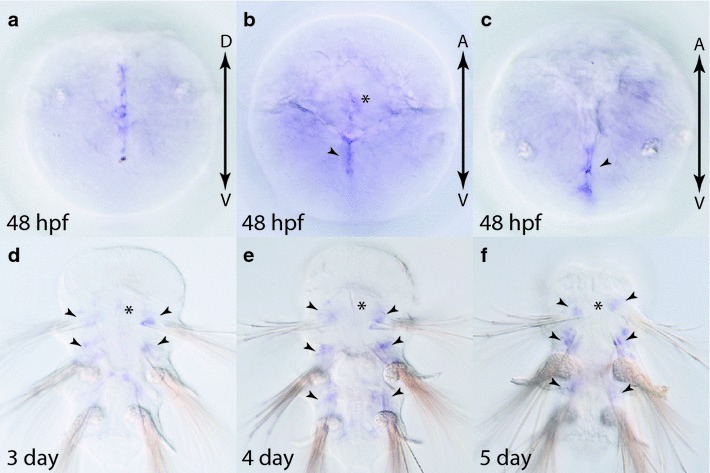
Expression of *sfrp3/4* in larval stages (48 hpf to 5-day old) in *Platynereis.* 48 hpf embryo from posterior (**a**), ventral (**b**) and dorsal (**c**) view. *Black arrowheads* indicate ventral and dorsal midlines. **d**–**f** 3 day, 4-day and 5-day larvae ventral view, anterior up. Gene expression analysis was performed with WMISH. Refer to the “Results” for details on the expression patterns. *Black arrowheads* indicate expression domains anterior to parapodia. *Black asterisks* indicate the stomodeum

Two Frizzled family genes, *fz4* and *sfrp3/4,* are not expressed in early embryos and 24 h larvae, but are expressed in older larvae (Figs. 12, 13). *fz4* is initially expressed throughout the trochophore at 48 hpf (Fig. 12a, e), and becomes more restricted to the head region and stomodeum by 72 hpf (Fig. 12b, f). At 4 and 5 days of development, expression of *fz4* becomes distinctly restricted to the stomodeum and ventral regions of the developing brain. In addition to anterior expression, *fz4* is also expressed within the second and third segments of the developing trunk (Fig. 12c, d, g, h).

*sfrp3/4* is the only frizzled family gene that was not detected in the head region or brain at any time during early and late development. Expression is first detectable at 48 hpf within the most posterior region and both the dorsal and ventral midlines (Fig. 13a–c). In addition to the midline expression, there appears to be weak expression in the stomodeum and trunk mesoderm (Fig. 13b). From 3 to 5 days of development, *sfrp3/4* is restricted to small bilaterally symmetric expression domains anterior to each of the parapodia (Fig. 13d–f).

## Discussion

### The ancestral lophotrochozoan *frizzled*-*related* gene complement

Our phylogenetic and structural analysis of the *frizzled*-*related* genes enabled the inference of the ancestral lophotrochozoan *frizzled*-*related* gene complement: four Frizzled receptors, *fz1/2/7, fz5/8, fz9/10, and fz4,* and two sFRPs*, sfrp1/2/5* and *sfrp3/4*. In agreement with previous studies [2, 17, 18], this ancestral complement was maintained from a eumetazoan, bilaterian, and protostome ancestor, and has been maintained in several extant invertebrate species within the deuterostome lineage (sea urchin and hemichordate), and three of the six lophotrochozoans (Table 1). A previous study showed that the ancestral eumetazoan *wnt* gene complement of 13 Wnt ligands was mostly retained within some extant deuterostomes (sea urchin *S.purpuratus* with 12 Wnts [24]) and some extant lophotrochozoans (*Platynereis* and *Capitella* with 12 Wnts; *Lottia* with 11 Wnts) [53, 54, 67]. This is significant, as it indicates that the morphological diversifications leading to most crown groups of the major bilaterian phyla happened without changes to the *frizzled*-*related* and *wnt* gene complements. In contrast, the morphological diversification of vertebrates was preceded by an increase from four to ten Frizzled receptors, from two to five sFRPs, and 12–19 Wnt ligands as a result of two whole genome duplications and subsequent gene loss at the base of the vertebrate lineage [2, 10, 19, 20]. However, during the major morphological diversification of vertebrate taxa since then, these gene complements were largely maintained.

### Modification to the *frizzled* gene complement within lophotrochozoans

Comparison of the six ancestral lophotrochozoan *frizzled*-*related* genes to six extant lophotrochozoans identifies species with conserved and derived *frizzled* genes. Of the three analyzed mollusks, *Crassostrea gigas* and *Aplysia californica* have retained all six *frizzled*-*related* genes, and *Lottia gigantea* has five genes and has lost *sfrp3/4*. However, most *frizzled*-*related* genes in *Aplysia* show stronger sequence divergence than any of the *Lottia* genes (Fig. 2). Of the three annelid species *Platynereis dumerilii* has retained all six, but has three additional genes that we interpret as gene duplicates of any of the six *frizzled*-*related* CRD domains. *Capitella* has retained five moderately conserved *frizzled*-*related* genes and lost the *sfrp3/4* gene. By far, the most derived gene set of the six lophotrochozoans was observed for the leech *Helobdella robusta* (loss of *fz4*, *fz5/8*, *sfrp3/4*, and duplications of *fz1/2/7*, *fz9/10*, and *sfrp1/2/5*). Again, morphological diversification within lophotrochozoan taxa is mostly not accompanied by changes to the *frizzled*-*related* gene complement. One exception is the leech *Helobdella*, which is regarded as a morphologically derived clitellate annelid [73], and which also exhibits a highly divergent *frizzled*-*related* gene set. It will be interesting to see whether a similar divergence can be observed in all clitellate species, or only in distinct sub-lineages.

### Divergence and loss of the *sfrp3/4* gene in lophotrochozoans

The *sfrp3/4* gene was the one of the six ancestral *frizzled*-*related* genes that experienced the most significant evolutionary changes within the lophotrochozoan lineages from loss in three species (*Lottia, Capitella,* and *Helobdella*) [17], high sequence derivation in two species (*Platynereis, Aplysia*), potential gene duplication in *Platynereis*, and strong conservation in *Crassostrea*. It will be interesting to see whether other lophotrochozoan species show a similar bias to evolutionary change for the *sfrp3/4* gene. Our phylogenetic and structural analysis identified *fzCRD*-*1* and a bona fide *sfrp3/4* gene as potential duplicates of an ancestral lophotrochozoan *sfrp3/4* gene in *Platynereis*. Interestingly, the CRD domain of *sfrp3/4* is highly derived, compared to the moderately conserved CRD domain of *fzCRD*-*1* that may indicate divergence in function of the two. Furthermore, we found strong expression of *fzCRD*-*1* in early embryonic lineages and strong expression in the head region of later stages, whereas the *sfrp3/4* gene was not expressed in embryonic stages and the head region but only in some trunk lineages. It is possible that both of these expression domains may represent functions of an ancestral *sfrp3/4* gene that were split in two after gene duplication as observed for other duplicated genes [74, 75].

### Novel *frizzled*-*related* genes in lophotrochozoans

Our study found several novel *frizzled*-*related* genes in lophotrochozoans that encode Fz-related CRD domains only (3 in *Platynereis*, 1 in *Aplysia*) that we interpret as more recent lineage-specific duplications from one of the six ancestral *frizzled*- *related* genes. These novel *frizzled*-*related* CRD genes resemble sFRPs in structure with an N-terminally located secretion signal and a potential Wnt ligand binding CRD domain, but lacking a C-terminal NTR domain. Especially the domains of FzCRD-1 in *Platynereis* are reminiscent structurally of a *fz4* splice variant in vertebrates that codes for a secreted protein consisting of only the N-terminal CRD of Fz4 and which can regulate Wnt signaling [72]. Therefore, these novel CRD genes might also function as modulators (antagonists or agonists) of Wnt signaling pathways. It is tempting to speculate that duplicates of *frizzled*-*related* CRD domains represent a frequently used toolbox during evolution to make cell populations inert to otherwise instructional Wnt signals and prevent certain cell fate changes.

### *Fz1/2/7* is a candidate for involvement in early beta-catenin-mediated binary cell fate decisions

One of the purposes of this study was to identify candidates among the *frizzled*- *related* genes that might be part of the molecular mechanism to orchestrate beta-catenin-mediated binary cell fate specification [48]. Based on the developmental RNA-seq time course and in situ hybridization, *fz1/2/7* emerged as the most likely candidate as its mRNA is maternally provided at high levels, and is inherited by all daughter cells during the first few rounds of cell division. High maternal contributions of *fz1/2/7* transcript have also been found in the cnidarian *C. hemispherica* and the echinoderm *P. lividus* [31, 32] suggesting that a function of *fz1/2/7* gene during the earliest stages of embryogenesis might be an evolutionarily conserved feature. In *P. lividus*, maternal *fz1/2/7* was also shown to be required for nuclear localization of beta-catenin protein [31]. Thus, *fz1/2/7* is an excellent candidate for future functional studies in *Platynereis.* However, even if *fz1/2/7* is directly involved in beta-catenin localization, it is not known through what molecular mechanism this could occur. A previous study of early *wnt* ligand expression in *Platynereis* revealed no obvious candidates or maternal contributions of any of the known *wnt* ligands, suggesting a Wnt ligand-independent mechanism for beta-catenin-mediated binary specification [54]. There is precedence for a mechanism like this in the nematode *C. elegans* where a similar global but highly derived beta-catenin-mediated binary specification mechanism has been described [51, 52]. Although every binary cell fate switch in *C. elegans* is dependent on a functional Frizzled receptor, many instances appear to be Wnt ligand independent [76]. The molecular mechanism underlying the Wnt ligand-independent beta-catenin-mediated binary cell fate specification in *C. elegans* remains largely unknown.

### An anterior Wnt antagonizing center in *Platynereis* embryos

During early embryogenesis in *Platynereis*, we found a dynamic expression of the *sfrp1/2/5* and *fz5/8* genes in the animal-pole cell lineages that will form the apical organ and the head region. Expression in the head region is also observed for both genes in early and late larval stages of *Platynereis,* consistent with a previous study [35]. These anterior expression domains are reminiscent of similar anterior territories expressing orthologous genes found in several other metazoans including cnidarian, cephalochordate, echinoderm, and hemichordate embryos and larvae [25–29, 77], and have been proposed to be part of an evolutionarily conserved anterior Wnt antagonizing signaling center in metazoans [3], to pattern anterior neuroectoderm in deuterostomes [33, 34, 78], and to constitute a developmental program to establish the apical territory and apical organ in invertebrates [35]. The restricted expression of these two genes in the most animal cell lineages early on may suggests that a similar Wnt antagonizing signaling center is being established during cleavage stages in *Platynereis* embryos.

### Early cell lineage expression of *frizzled*-*related* genes predicts expression domains in later larvae

The five *frizzled*-*related* genes transcribed during early embryogenesis in *Platynereis*, *fz1/2/7*, *fz9/10*, *fz5/8*, *sfrp1/2/5, and fzCRD*-*1*, are all expressed in the four most animal cells (1q^11^) and their progeny. 1q^11^ cells are born at the ~32-cell stage and will divide to generate hundreds of cells that will form the entire head region including the eyes, brain structures and apical organ of later larval stages [44, 45]. Intriguingly, all five of these genes continue to be expressed prominently in the anterior head region of early and late larval stages. Two, *fz1/2/7* and *fz9/10,* exhibit an additional and prominent early expression domain in the 2d cell lineage that will give rise to the trunk ectoderm of the larvae [45]. Remarkably, these are the only two *frizzled* genes that are prominently expressed throughout the trunk region in later larval stages. Thus, *frizzled*-*related* genes appear to maintain cell lineage restricted expression domains from embryo to larval stages. Similar lineage restrictions have been observed for embryonic and larval expression of *wnt* ligands in *Platynereis* embryos [54]. Whether these lineage restrictions indicate that potential embryonic polarities and signal receiving territories established by *frizzled*-*related* genes are maintained through larval stages or whether they support separate embryonic and larval functions remains to be determined.

### *Frizzled*-*related* gene expression is biased towards anterior expression

Overall we observed a preference for anterior expression of *frizzled*-*related* genes in embryonic lineages that extends through larval stages with prominent expression domains of *fz1/2/7*, *fz9/10*, *fz5/8*, *fz4*, *sfrp1/2/5*, and the possibly derived *sfrp3/4*-*related* gene *fzCRD*-*1* in the head region of larval stages (Figs. 11, 12). This is in contrast to our previous study of the 12 *wnt* ligands in *Platynereis* that are predominantly expressed in various posterior domains in the trunk region of larvae (9 of 12 *wnts*), and only sparsely in the head region (4 of 12 *wnts*) [54]. Thus, the majority of Wnt secreting cells are localized in posterior domains, while the majority of cells expressing *frizzled*-*related* genes and capable to receive, modulate, or inhibit Wnt signals are located in anterior territories of embryo and larvae. Wnt signaling is intimately tied to the early establishment of embryonic polarity and axis formation in many metazoan embryos [3, 79] with posterior expression of selected *wnt* ligands, and anterior expression of selected *frizzleds* and *sfrps* observed in several taxa. The use of posterior Wnt signaling and anterior Wnt inhibition has been proposed as a ‘unifying principle of body plan development in animals’ [3]. Thus, the observed bias in expression of *frizzled*-*related* genes anteriorly and of *wnt* ligands posteriorly in *Platynereis* might be the evolutionary remnants and products of an ancient mechanism to pattern metazoan embryos along the anterior–posterior axis.

## Conclusions

We present the first analysis of *frizzled*-*related* genes in lophotrochozoans, and the first comprehensive report of *frizzled* gene expression during spiral development and larval stages of a member of the lophotrochozoans, the annelid *Platynereis dumerilii*. We have determined that *Platynereis* and other lophotrochozoans retained an overall well-conserved set of *frizzled* and *sfrp* genes. High maternal expression identifies *fz1/2/7* as the only *frizzled* gene to be in the right place at the right time for Wnt signaling functions during early cleavage stages. *sfrp1/2/5* and *fz5/8* are expressed in the most anterior cell lineages suggesting evolutionarily conserved roles in the formation of an anterior Wnt antagonizing center in this annelid. In general, *frizzled*-*related* genes show a bias towards anterior expression in early embryos and larval stages. This study provides new insights into the role of Frizzleds in Wnt signaling in a spiral-cleaving embryo and annelid larval stages, has identified numerous regions with competence to receive and/or modulate Wnt signals, and suggests the existence of an evolutionary conserved patterning system along the anterior–posterior axis of this annelid. Therefore, this study uncovered many potential Wnt signaling activities during *Platynereis* development, and sets the stage for a functional dissection of specific roles of this pathway in cell fate specification and patterning in this lophotrochozoan species.

## Additional files

10.1186/s13227-015-0032-4 Identifiers for Frizzed-related proteins of species used for Fig. 2 and Table 1. Identifiers and accession numbers are shown for each Frizzled-related sequence that was used in various phylogenetic analyses in this study. For the analysis presented in Fig. 2 all Frizzled-related sequences were included from species (1) that represent each of the major animal branches, and (2) that in general retained an ancestral gene complement. Sequences with an asterisk were removed from the phylogenetic analysis (1) to restrict the total number of sequences shown, or (2) to remove sequences that were very divergent and had an adverse effect on the analysis e.g. long branch attraction. However, the annotations shown here are well supported by additional phylogenetic analyses (data not shown). Lophotrochozoan sequences are highlighted in red. The majority of protein sequences were obtained from NCBI, Bf_Fz5/8 and Ct_sFRP1/2/5 from the Joint Genome Institute, and Sk_Fz9/10 and Dr_sFRP2L were translated from mRNA sequences obtained from NCBI. Gene names for *D. melanogaster* and *C. elegans* are given in parentheses. Species abbreviations: Ac, *Aplysia californica*; Bf, *Branchiostoma floridae*; Ce, *Caenorhabditis elegans*; Cg, *Crassostrea gigas*; Ct, *Capitella teleta*; Dm, *Drosophila melanogaster*; Dp, *Daphnia pulex*; Dr, *Danio rerio*; Hr, *Helobdella robusta*; Hs, *Homo sapiens*; Lg, *Lottia gigantea*; Nv, *Nematostella vectensis*; Sk, *Saccoglossus kowalevskii*; Sp, *Strongylocentrotus purpuratus*; Tc, *Tribolium castaneum*; Xl, *Xenopus laevis.*

10.1186/s13227-015-0032-4 Phylogenetic Analysis of Netrin domain containing proteins identifies PdsFRP3/4 and PdsFRP1/2/5, and indicates independent origins for the two sFRP gene families. Netrin domains of sFRPs and other NTR domain containing proteins were aligned in MAFFT and analyzed with Mr. Bayes. *Nematostella vectensis* TIMP was used as an outgroup. Posterior probabilities greater than 70 % are shown. *P. dumerilii* proteins are highlighted in red. *P. dumerilii* sFRP3/4 clusters with other sFRP3/4 s with high posterior probability confirming its identification as an sFRP3/4 homolog despite its highly derived CRD. The sFRP1/2/5 and sFRP3/4 subfamilies are highlighted with green and blue boxes, respectively. Species abbreviations: Bf, *Brachiostoma floridae*; Cg, *Crassostrea gigas*; Ct, *Capitella teleta*; Dr, *Danio rerio;* Gg, *Gallus gallus;* Hs, *Homo sapiens;* Nv, *Nematostella vectensis;* Pd, *Platynereis dumerilii;* Sk, *Saccoglossus kowalevskii*; Sp, *Strongylocentrotus purpuratus;* Xl, *Xenopus laevis.*

10.1186/s13227-015-0032-4 Netrin domain containing proteins used for NTR phylogeny. Identifiers and accession numbers are given for each NTR domain containing protein. All sequences are protein sequences obtained from NCBI except Dr_sFRP2L that was translated from an mRNA sequence from NCBI. Species abbreviations: Bf, *Brachiostoma floridae*; Cg, *Crassostrea gigas*; Ct, *Capitella teleta*; Dr, *Danio rerio;* Gg, *Gallus gallus;* Hs, *Homo sapiens;* Nv, *Nematostella vectensis;* Pd, *Platynereis dumerilii;* Sk, *Saccoglossus kowalevskii*; Sp, *Strongylocentrotus purpuratus;* Xl, *Xenopus laevis.*

10.1186/s13227-015-0032-4 RNA-seq data for each of the nine *frizzled related* genes during early development of *Platynereis*. Quantitative expression levels are shown as FPKM for each gene at two-hour time points from 2 to 14 hpf (related to Fig. 4B–F). Independent measurements for two biological replicates are shown with higher values in the gray rows and lower values in white rows.
10.1186/s13227-015-0032-4 Expression of *sfrp1/2/5* during early development of *Platynereis*. Additional side views of (A-D) WMISH of *sfrp1/2/5,* and (A’-D’) false color images of WMISH (red) overlain with DAPI stained nuclear images (blue) in 10 hpf embryos (related to Fig. 9D-D''). (A, A’) view of the A quadrant, (B, B’) view of the B quadrant. (C, C’) view of the C quadrant, and (D, D’) view of the D quadrant. All images are oriented with the animal pole up. Asterisks indicate animal pole. Double arrows indicate the orientation of the animal-vegetal axis (A-V).
10.1186/s13227-015-0032-4 Expression of *fzCRD*-*1* in late trochophore larvae (48hpf) of *Platynereis*. (A) Anterior view with dorsal side up; waning expression of *fzCRD*-*1* in the ring canal (black arrowheads). (B) Dorsal view with anterior side up. Black arrowheads indicate bilaterally symmetric domains that may indicate the site where the developing chaetae erupt from the embryo. Double arrows indicate the orientation of the dorsal–ventral (D-V) and animal-vegetal (A-V) axes. Gene expression analysis was performed with WMISH (see also Fig. 10).

## Abbreviations

BLAST: Basic Local Alignment Search Tool
CRD: Cysteine-Rich Domain
DAPI: 4′,6-diamidino-2-phenylindole
FPKM: Fragments Per Kilobase per Million mapped reads
Fz: Frizzled
hpf: Hours post fertilization
sFRP: Secreted frizzled-related protein
nt: Nucleotide
NTR: Netrin
WMISH: Whole-mount in situ hybridization
