# Supplementary material for: Structure, phylogeny, and expression of the frizzled-related gene family in the lophotrochozoan annelid Platynereis dumerilii
Source: EvoDevo. 2015 Dec 4;6:37. doi: 10.1186/s13227-015-0032-4 (PMC4669655; doi:10.1186/s13227-015-0032-4)
Supplement: Supplementary file 1 — 10.1186/s13227-015-0032-4 Identifiers for Frizzed-related proteins of species used for Fig. 2 and Table 1. Identifiers and accession numbers are shown for each Frizzled-related sequence that was used in various phylogenetic analyses in this study. For the analysis presented in Fig. 2 all Frizzled-related sequences were included from species (1) that represent each of the major animal branches, and (2) that in general retained an ancestral gene complement. Sequences with an asterisk were removed from the phylogenetic analysis (1) to restrict the total number of sequences shown, or (2) to remove sequences that were very divergent and had an adverse effect on the analysis e.g. long branch attraction. However, the annotations shown here are well supported by additional phylogenetic analyses (data not shown). Lophotrochozoan sequences are highlighted in red. The majority of protein sequences were obtained from NCBI, Bf_Fz5/8 and Ct_sFRP1/2/5 from the Joint Genome Institute, and Sk_Fz9/10 and Dr_sFRP2L were translated from mRNA sequences obtained from NCBI. Gene names for D. melanogaster and C. elegans are given in parentheses. Species abbreviations: Ac, Aplysia californica; Bf, Branchiostoma floridae; Ce, Caenorhabditis elegans; Cg, Crassostrea gigas; Ct, Capitella teleta; Dm, Drosophila melanogaster; Dp, Daphnia pulex; Dr, Danio rerio; Hr, Helobdella robusta; Hs, Homo sapiens; Lg, Lottia gigantea; Nv, Nematostella vectensis; Sk, Saccoglossus kowalevskii; Sp, Strongylocentrotus purpuratus; Tc, Tribolium castaneum; Xl, Xenopus laevis. [file 13227_2015_32_MOESM1_ESM.pdf]

Frizzled proteins	Accesion number/ref. genome
**Frizzled 1/2/7**	
Ac_Fz1/2/7	XP_005103676
*Bf_Fz1/2/7	XP_002605403
*Ce_Fz1/2/7 (MOM-5)	NP_492635
Cg_Fz1/2/7	XP_011422642
Ct_Fz1/2/7	ELT94235
*Dm_Fz1/2/7 (Fz)	NP_001261836
Dp_Fz1/2/7	EFX86266
Dr_Fz1	NP_001124086
Dr_Fz2	NP_571215
Dr_Fz7a	AAH56280
Dr_Fz7b	AAH49397
Hr_Fz1/2/7a	XP_009009899
Hr_Fz1/2/7b	XP_009014585
Hs_Fz1	NP_003496
Hs_Fz2	NP_001457
Hs_Fz7	NP_003498
Lg_Fz1/2/7	XP_009047666
Nv_Fz1/2/7	XP_001647540
Sk_Fz1/2/7	XP_006820151
*Sp_Fz1/2/7	XP_003724561
Tc_Fz1/2/7	NP_001164247
Xl_Fz1	NP_001079207
Xl_Fz2	NP_001083829
Xl_Fz7	CAB45875
**Frizzled 4**	
Ac_Fz4	XP_005112462
*Bf_Fz4	XP_002612241
*Ce_MIG-1 (Fz4)	NP_490796
Cg_Fz4	EKC19233
Ct_Fz4	ELU03627
*Dm_Fz4	AAF81195
*Dp_Fz4	EFX79362
Dr_Fz4	NP_001292398
Hs_Fz4	NP_036325
Lg_Fz4	XP_009050162
Nv_Fz4	XP_001622965
Sk_Fz4	XP_002730495
*Sp_Fz4	XP_011682220
Tc_Fz4	XP_967603
Xl_Fz4	NP_001083922
**Frizzled 5/8**	
Ac_Fz5/8	XP_005093188
*Bf_Fz5/8	estExt_fgenesh2_pg.C_1280051
*Ce_Fz5/8 (CFZ-2)	NP_503965
Cg_Fz5/8	XP_011451646
Ct_Fz5/8	ELT92800
*Dm_Fz5/8 (Fz2)	AAC47273
Dp_Fz5/8	EFX87639
Dr_Fz5	NP_571209
Dr_Fz8a	NP_570993
Dr_Fz8b	NP_571628
Hs_Fz5	NP_003459
Hs_Fz8	NP_114072
Lg_Fz5/8	XP_009048933
Nv_Fz5/8	XP_001634995
Sk_Fz5/8	NP_001161547
*Sp_Fz5/8	XP_003724546
Tc_Fz5/8	XP_968118
Xl_Fz5	NP_001079217
Xl_Fz8	AAI69949
**Frizzled 9/10**	
Ac_Fz9/10	XP_005100653
*Bf_Fz9/10	XP_002599055
*Ce_Fz9/10 (LIN-17)	NP_001021813
Cg_Fz9/10	XP_011456333
Ct_Fz9/10	ELU07504

Frizzled proteins	Accesion number/ref. genome
*Dm_Fz9/10 (Fz3)	BAA84677
Dr_Fz9a	CAQ14916
Dr_Fz9b	XP_003198734
Dr_Fz10	CAD10102
Hr_Fz9/10	XP_009027738
Hr_Fz9/10b	XP_009028364
Hs_Fz9	AAH26333
Hs_Fz10	AAH70037
Lg_Fz9/10	XP_009060024
Nv_Fz9/10	XP_001630630
Sk_Fz9/10	XM_006817125 from mRNA
*Sp_Fz9/10	XP_011664398
Xl_Fz10a	AAI69887
Xl_Fz10b	AAH72128
**sFRP 1/2/5**	
Ac_sFRP1/2/5	NP_001191650
*Bf_sFRP1/2/5a	XP_002608045
*Bf_sFRP1/2/5b	XP_002611318
*Ce_SFRP-1 (sFRP1/2/5)	NP_500977
Cg_sFRP1/2/5	XP_011456955
Ct_sFRP1/2/5	e_gw1.111.17.1
Dp_sFRP1/2/5	EFX90356
Dr_sFRP1a	AAI08048
Dr_sFRP1b	NP_001077040
Dr_sFRP2	AAI24708
Dr_sFRP2L	XM_003200104 from mRNA
Dr_sFRP5	NP_571933
*Dr_Szl	AAH94990
Hr_sFRP1/2/5a	XP_009023421
Hr_sFRP1/2/5b	XP_009028340
Hs_sFRP1	NP_003003
Hs_sFRP2	NP_003004
Hs_sFRP5	AAD25052
Lg_sFRP1/2/5	XP_009051836
Nv_sFRP1/2/5	XP_001638620
Sk_sFRP1/2/5	NP_001161656
*Sp_sFRP1/2/5	XP_781087
*Xl_Crescent	NP_001082025
Xl_sFRP1	NP_001080957
Xl_sFRP2	NP_001080663
Xl_sFRP5	NP_001083946
*Xl_Szl	NP_001081990
**sFRP 3/4**	
Ac_sFRP3/4	XP_005110283
Bf_sFRP3/4	XP_002612882
Cg_sFRP3/4	EKC37416
Dp_sFRP3/4	EFX72724
Dr_sFRP3/4	AJG06035
Hs_sFRP3	AAC50736
Hs_sFRP4	CAG46532
Nv_sFRP3/4	XP_001638660
Sk_sFRP3/4	XP_006825063
*Sp_sFRP3/4	XP_011667363
Xl_sFRP3/4	AAC60113
**Smoothened**	
Ac_Smo	XP_005106761
Cg_Smo	XP_011413301
Ct_Smo	ELT97156
*Dm_Smo	NP_523443
Dp_Smo	EFX80809
Dr_Smo	AAK83380
Hr_Smo	XP_009031206
Hs_Smo	NP_005622
Lg_Smo	XP_009064048
Nv_Smo	XP_001632182
Sk_Smo	XP_006817784
Tc_Smo	NP_001127850
Xl_Smo	NP_001082084

Additional File 1
