# Supplementary figures and images for: Structure, phylogeny, and expression of the frizzled-related gene family in the lophotrochozoan annelid Platynereis dumerilii
Source: EvoDevo. 2015 Dec 4;6:37. doi: 10.1186/s13227-015-0032-4 (PMC4669655; doi:10.1186/s13227-015-0032-4)

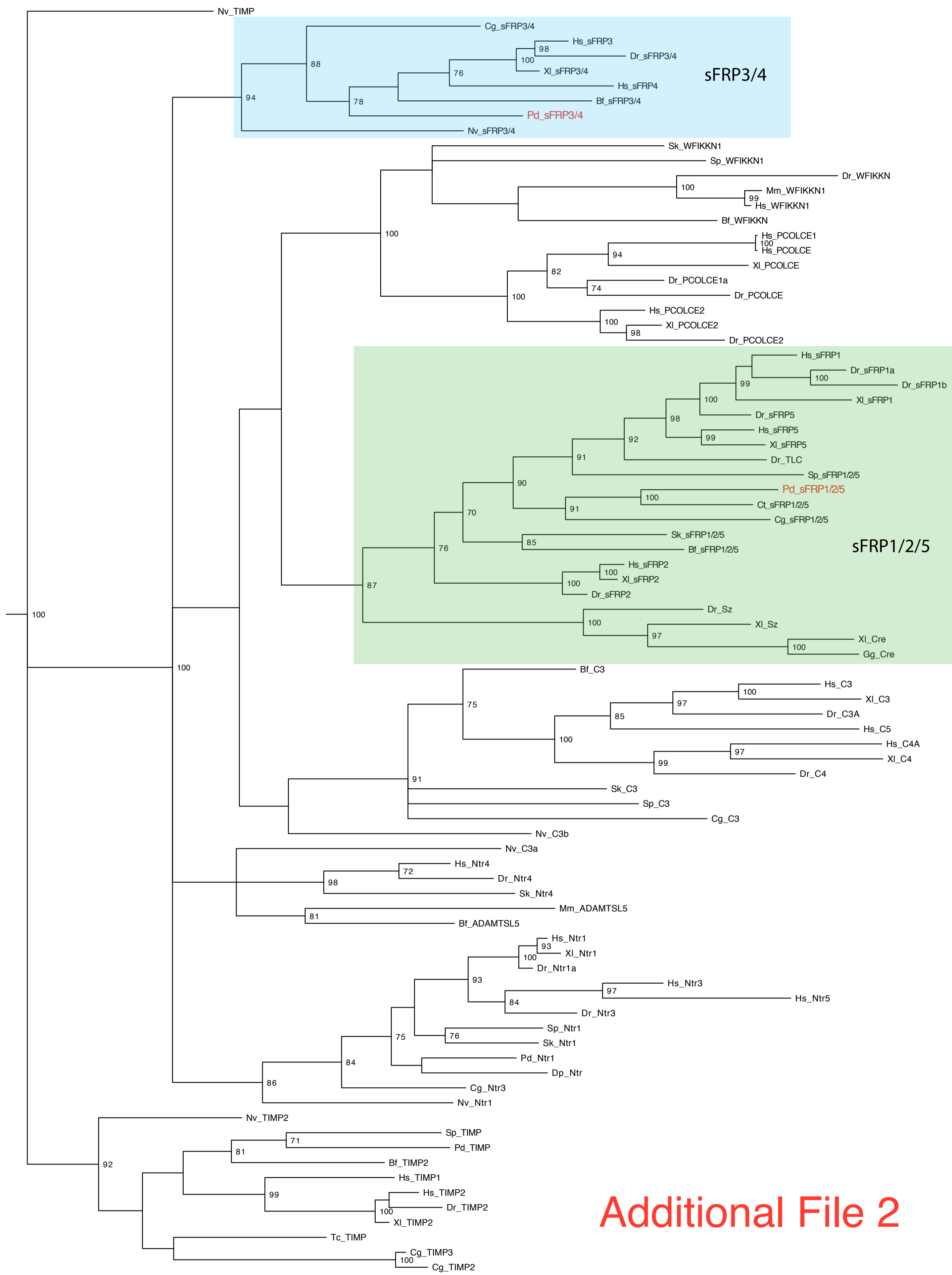

Additional File 2

Supplement: Supplementary file 2 — 10.1186/s13227-015-0032-4 Phylogenetic Analysis of Netrin domain containing proteins identifies PdsFRP3/4 and PdsFRP1/2/5, and indicates independent origins for the two sFRP gene families. Netrin domains of sFRPs and other NTR domain containing proteins were aligned in MAFFT and analyzed with Mr. Bayes. Nematostella vectensis TIMP was used as an outgroup. Posterior probabilities greater than 70 % are shown. P. dumerilii proteins are highlighted in red. P. dumerilii sFRP3/4 clusters with other sFRP3/4 s with high posterior probability confirming its identification as an sFRP3/4 homolog despite its highly derived CRD. The sFRP1/2/5 and sFRP3/4 subfamilies are highlighted with green and blue boxes, respectively. Species abbreviations: Bf, Brachiostoma floridae; Cg, Crassostrea gigas; Ct, Capitella teleta; Dr, Danio rerio; Gg, Gallus gallus; Hs, Homo sapiens; Nv, Nematostella vectensis; Pd, Platynereis dumerilii; Sk, Saccoglossus kowalevskii; Sp, Strongylocentrotus purpuratus; Xl, Xenopus laevis. [file 13227_2015_32_MOESM3_ESM.pdf]

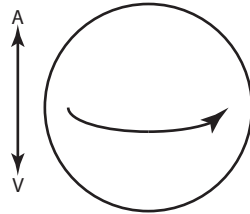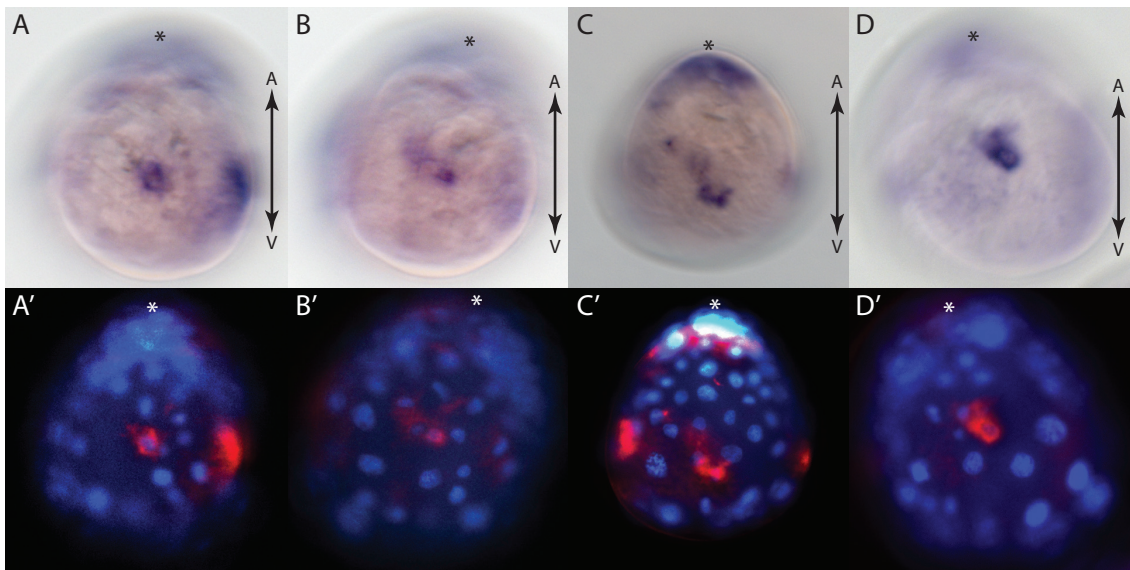

Supplement: Supplementary file 5 — 10.1186/s13227-015-0032-4 Expression of sfrp1/2/5 during early development of Platynereis. Additional side views of (A-D) WMISH of sfrp1/2/5, and (A’-D’) false color images of WMISH (red) overlain with DAPI stained nuclear images (blue) in 10 hpf embryos (related to Fig. 9D-D''). (A, A’) view of the A quadrant, (B, B’) view of the B quadrant. (C, C’) view of the C quadrant, and (D, D’) view of the D quadrant. All images are oriented with the animal pole up. Asterisks indicate animal pole. Double arrows indicate the orientation of the animal-vegetal axis (A-V). [file 13227_2015_32_MOESM5_ESM.pdf]

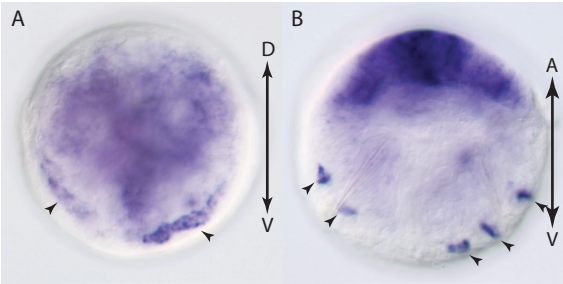

Supplement: Supplementary file 6 — 10.1186/s13227-015-0032-4 Expression of fzCRD-1 in late trochophore larvae (48hpf) of Platynereis. (A) Anterior view with dorsal side up; waning expression of fzCRD-1 in the ring canal (black arrowheads). (B) Dorsal view with anterior side up. Black arrowheads indicate bilaterally symmetric domains that may indicate the site where the developing chaetae erupt from the embryo. Double arrows indicate the orientation of the dorsal–ventral (D-V) and animal-vegetal (A-V) axes. Gene expression analysis was performed with WMISH (see also Fig. 10). [file 13227_2015_32_MOESM6_ESM.pdf]
