# Supplementary material for: Structure, phylogeny, and expression of the frizzled-related gene family in the lophotrochozoan annelid Platynereis dumerilii
Source: EvoDevo. 2015 Dec 4;6:37. doi: 10.1186/s13227-015-0032-4 (PMC4669655; doi:10.1186/s13227-015-0032-4)
Supplement: Supplementary file 3 — 10.1186/s13227-015-0032-4 Netrin domain containing proteins used for NTR phylogeny. Identifiers and accession numbers are given for each NTR domain containing protein. All sequences are protein sequences obtained from NCBI except Dr_sFRP2L that was translated from an mRNA sequence from NCBI. Species abbreviations: Bf, Brachiostoma floridae; Cg, Crassostrea gigas; Ct, Capitella teleta; Dr, Danio rerio; Gg, Gallus gallus; Hs, Homo sapiens; Nv, Nematostella vectensis; Pd, Platynereis dumerilii; Sk, Saccoglossus kowalevskii; Sp, Strongylocentrotus purpuratus; Xl, Xenopus laevis. [file 13227_2015_32_MOESM2_ESM.pdf]

Netrin domain proteins	Accession numbers
**sFRP1/2/5**	
Bf_sFRP1/2/5	XP_002608045
Cg_sFRP1/2/5	XP_011456955
Ct_sFRP1/2/5	e_gw1.111.17.1
Dr_sFRP1a	AAI08048
Dr_sFRP1b	NP_001077040
Dr_sFRP2	AAI24708
Dr_sFRP2L	XM_003200104 from mRNA
Dr_sFRP5	NP_571933
Dr_Sz	AAH94990
Dr_TLC	NP_938176
Gg_Cre	NP_990430
Hs_sFRP1	NP_003003
Hs_sFRP2	NP_003004
Hs_sFRP5	AAD25052
Nv_sFRP1/2/5	XP_001638620
Sk_sFRP1/2/5	NP_001161656
Sp_sFRP1/2/5	XP_781087
Xl_Cre	NP_001082025
Xl_sFRP1	NP_001080957
Xl_sFRP2	NP_001080663
Xl_sFRP5	NP_001083946
Xl_Sz	NP_001081990
**sFRP3/4**	
Bf_sFRP3/4	XP_002612882
Cg_sFRP3/4	EKC37416
Dr_sFRP3/4	AJG06035
Hs_sFRP3	AAC50736
Hs_sFRP4	CAG46532
Nv_sFRP3/4	XP_001638660
Xl_sFRP3/4	AAC60113

Netrin domain proteins	Accession numbers
**Other NTR domain proteins**	
Bf_ADAMTSL5	XP_002586752
Bf_C3	XP_002612866
Bf_TIMP2	AEU03843
Bf_WFIKKN	XP_002607180
Cg_C3	EKC24393
Cg_Ntr3	EKC26179
Cg_TIMP2	NP_001292265
Cg_TIMP3	NP_001292268
Dp_Ntr	EFX75164
Dr_C3A	NP_571317
Dr_C4	XP_001334640
Dr_Ntr1a	NP_571104
Dr_Ntr3	NP_001032492
Dr_Ntr4	AAI63582
Dr_PCOLCE	NP_001122259
Dr_PCOLCE1a	NP_001025352
Dr_PCOLCE2	XP_009295891
Dr_TIMP2	NP_878294
Dr_WFIKKN	AAH68412
Hs_C3	NP_000055
Hs_C4A	NP_001239133
Hs_C5	NP_001726
Hs_Ntr1	AAD09221
Hs_Ntr3	NP_006172
Hs_Ntr4	NP_067052
Hs_Ntr5	NP_665806
Hs_PCOLCE	EAL23825
Hs_PCOLCE1	EAL23825
Hs_PCOLCE2	NP_037495
Hs_TIMP1	EAW59316
Hs_TIMP2	EAW89539
Hs_WFIKKN1	NP_444514
Mm_ADAMTSL5	EDL31560
Mm_WFIKKN1	AAH26460
Nv_C3a	BAH22725
Nv_C3b	BAH22723
Nv_Ntr1	XP_001628899
Nv_TIMP	XP_001625448
Nv_TIMP2	XP_001625449
Pd_Ntr1	ABO93214
Sk_C3	XP_006823058
Sk_Ntr1	NP_001158400
Sk_Ntr4	NP_001164710
SkWFIKKN1	XP_002732704
Sp_C3	NP_999686
Sp_Ntr1	XP_011669599
Sp_TIMP	XP_780837
Sp_WFIKKN	XP_011672335
Xl_C3	2116268A
Xl_C4	AAI70422
Xl_Ntr1	AAB87983
Xl_PCOLCE	NP_001087777
Xl_PCOLCE2	NP_001080499
Xl_TIMP2	NP_001087748

Additional File 3
