# Supplementary material for: Structure, phylogeny, and expression of the frizzled-related gene family in the lophotrochozoan annelid Platynereis dumerilii
Source: EvoDevo. 2015 Dec 4;6:37. doi: 10.1186/s13227-015-0032-4 (PMC4669655; doi:10.1186/s13227-015-0032-4)
Supplement: Supplementary file 4 — 10.1186/s13227-015-0032-4 RNA-seq data for each of the nine frizzled related genes during early development of Platynereis. Quantitative expression levels are shown as FPKM for each gene at two-hour time points from 2 to 14 hpf (related to Fig. 4B–F). Independent measurements for two biological replicates are shown with higher values in the gray rows and lower values in white rows. [file 13227_2015_32_MOESM4_ESM.pdf]

Gene	2hpf	4hpf	6hpf	8hpf	10hpf	12hpf	14hpf
Fz1/2/7	103.5	20.0	17.5	12.1	53.6	57.2	18.5
	85.4	17.1	16.2	9.0	53.4	38.9	12.7
Fz4	0.1	0.1	0.3	0.3	0.5	0.2	0.4
	0.1	0.1	0.2	0.2	0.4	0.2	0.3
Fz5/8	0.0	3.3	8.9	4.5	13.0	14.2	5.3
	0.0	0.1	6.8	3.5	10.8	11.5	3.6
Fz9/10	1.7	1.0	4.6	8.7	8.2	4.1	7.5
	0.3	0.5	2.1	6.0	4.9	4.1	5.5
sFRP1/2/5	0.0	0.0	1.4	16.8	17.5	8.9	8.5
	0.0	0.0	0.4	13.2	13.3	7.0	8.3
sFRP3/4	0.1	0.1	0.3	0.2	0.2	0.1	0.0
	0.1	0.0	0.1	0.2	0.1	0.1	0.0
FzCRD-1	0.4	0.7	0.6	1.0	9.6	17.8	17.1
	0.2	0.7	0.6	0.2	6.4	13.2	16.9
FzCRD-2	0.1	0.0	0.0	0.0	0.0	0.1	0.2
	0.0	0.0	0.0	0.0	0.0	0.0	0.0
FzCRD-3	0.1	0.0	0.0	0.1	0.0	0.1	0.0
	0.0	0.0	0.0	0.0	0.0	0.0	0.0

Additional File 4
